# New insights into freshwater ascomycetes: discovery of novel species in diverse aquatic habitats

**DOI:** 10.3389/fcimb.2024.1515972

**Published:** 2025-01-13

**Authors:** Lu Li, Darbhe Jayarama Bhat, Hong-Bo Jiang, Jun-Fu Li, Turki M. Dawoud, Fangqi Sun, Sukanya Haituk, Ratchadawan Cheewangkoon, Rungtiwa Phookamsak

**Affiliations:** ^1^ Department of Entomology and Plant Pathology, Faculty of Agriculture, Chiang Mai University, Chiang Mai, Thailand; ^2^ Agrobiodiversity in Highland Agriculture and Sustainable Utilization Research Group, Chiang Mai University, Chiang Mai, Thailand; ^3^ Key Laboratory of Phytochemistry and Natural Medicines, Kunming Institute of Botany, Chinese Academy of Sciences, Kunming, Yunnan, China; ^4^ Department of Botany and Microbiology, College of Science, King Saud University, Riyadh, Saudi Arabia; ^5^ Vishnugupta Vishwavidyapeetam, Gokarna, India; ^6^ Department of Economic Plants and Biotechnology, Yunnan Key Laboratory for Wild Plant Resources, Kunming Institute of Botany, Chinese Academy of Sciences, Kunming, Yunnan, China; ^7^ Honghe Center for Mountain Futures, Kunming Institute of Botany, Chinese Academy of Sciences, Yunnan, China; ^8^ Centre for Mountain Futures (CMF), Kunming Institute of Botany, Chinese Academy of Sciences, Kunming, Yunnan, China; ^9^ CIFOR-ICRAF China Program, World Agroforestry (ICRAF), Kunming, China; ^10^ Office of the Research Administration, Chiang Mai University, Chiang Mai, Thailand

**Keywords:** Fuscosporellaceae, hyphomycetes, morpho-molecular-based taxonomy, Nectriaceae, novel taxa, Pleurotheciaceae

## Abstract

During investigations of freshwater fungi in Hunan and Yunnan provinces, China, *Chaetopsina yunnanensis* sp. nov. (Nectriaceae), *Parafuscosporella hunanensis* sp. nov. (Fuscosporellaceae), and *Pleurotheciella yunnanensis* sp. nov. (Pleurotheciaceae) were discovered on submerged decaying wood and branches. Based on phylogenetic analyses, *C. yunnanensis* formed a separate branch with *Chaetopsina pinicola* and nested among other *Chaetopsina* species in Nectriaceae (Hypocreales). Furthermore, hitherto known *Chaetopsina beijingensis* shared the same branch with *Chaetopsina fulva*, a type species of the genus, demonstrating their conspecific status. Therefore, *C. beijingensis* is formally synonymized under *C. fulva*, with an amended species circumscription. *Pa. hunanensis* formed a well-separated subclade with the ex-type strain of *Parafuscosporella mucosa* and clustered with other *Parafuscosporella* within Fuscosporellaceae (Fuscosporellales). In addition, the genus *Parafuscosporella* is treated as distinct from *Vanakripa* due to a lack of phylogenetic evidence in clarifying their congeneric status with the latter. *Pl. yunnanensis* is found to be sister to *Pleurotheciella saprophytica*, forming a subclade with *Pleurotheciella dimorphospora* within the Pleurotheciaceae (Pleurotheciales). Morphologically, *C. yunnanensis* fits well with the generic concept of *Chaetopsina* in forming a holomorphic state with hyphomycetous asexual morph producing pigmented, setiform conidiophores, phialidic conidiogenous cells, hyaline conidia, and nectria-like sexual morph. *Pa. hunanensis* fits well with *Parafuscosporella* in having acrogenous, apiosporous, versicolored, obovoid to obpyriform conidia. In contrast, *Pl. yunnanensis* resembles *Pl. dimorphospora* in forming asexual dimorphism with two types of conidia (Type I, brown, muriform/phragmosporous conidia; Type II, hyaline, amerosporous/didymorsporous conidia). The novelty of taxa is explained with detailed descriptions, photo-micrographic illustrations, polymorphism, and multigene phylogenetic analyses of Bayesian inference and maximum likelihood criteria.

## 1 Introduction

Freshwater fungi are a diverse and heterogeneous taxonomic group occurring saprobically on partially or fully submerged organic substrates in aquatic habitats. Their life cycle, in whole or part, relies on free freshwater and submerged substrates (Calabon et al., 2020, 2023a). Freshwater environments are categorized into three types: 1) lentic, any natural aquatic environment lacking continuous flow but exhibiting static, low, or slow movement of water as in lakes, ponds, swamps, and pools; 2) lotic, any natural aquatic environment with a continuous flow of water such as rivers, streams, creeks, and brooks; and 3) other habitats, which include artificial water bodies such as in cooling towers and tree holes (Luo et al., 2004). Fungi found in freshwater habitats were grouped into several morphological and ecological entities, viz., freshwater ascomycetes, freshwater hyphomycetes (i.e., Ingoldian fungi, aero-aquatic hyphomycetes or asexual ascomycetes, terrestrial–aquatic hyphomycetes, and submerged aquatic hyphomycetes), freshwater basidiomycetes, coelomycetes, microsporidia, zoosporic fungi, and zygomycetes (Tsui et al., 2016; Schuster et al., 2022). Furthermore, freshwater fungi subsist as saprobes, mutualists, or parasites and have also been isolated as endophytes (Tsui et al., 2016). However, freshwater fungi have an important ecological function in decomposing wood by breaking down complex organic compounds into simpler inorganic materials and facilitating the passage of energy and nutrients across all trophic levels in the food chain (Bärlocher et al., 2011; Sridhar and Sudheep, 2011; Tsui et al., 2016). Two crucial biological characteristics of these fungi include the ability to sporulate underwater and thrive on decaying deciduous leaves and twigs in streams and rivers (Krauss et al., 2011; Wurzbacher et al., 2014; Tsui et al., 2016). In addition, the biotechnological potential of freshwater fungi as producers of bioactive metabolites, with promising values in drug discovery, is becoming evident (Duarte et al., 2013; Shen et al., 2022; El-Elimat et al., 2021; Calabon et al., 2023a). Lignicolous freshwater fungi can degrade indigestible lignocellulose in submerged wood, releasing nutrients into the water. However, their precise ecological role and economic value remain less understood (Bucher et al., 2004; Shen et al., 2022).

Freshwater fungi have been documented since the mid-19th century, and a wealth of information is now available (de Wildeman, 1895; Ingold, 1942, 1955; Calabon et al., 2021). Over the recent past decades, these fungi have been relatively well-studied in Asia, particularly in China, India, and Thailand (Sridhar and Sudheep, 2011; Mehboob et al., 2021; Luo et al., 2019; Dong et al., 2020; Shen et al., 2022; Calabon et al., 2023a, b). In the early stages of studies on freshwater fungi, identification has primarily relied on morphology (Ingold, 1975; Krauss et al., 2011; Calabon et al., 2023a). In the case of yeasts, additional methods such as biochemical, fermentation, and assimilation tests were included. Presently, in addition to morphology, molecular data analyses were applied to taxonomic studies of freshwater fungi (Ranghoo et al., 1999; Nikolcheva and Bärlocher, 2002; Luo et al., 2019; Dong et al., 2020; Calabon et al., 2023a, b). Recent studies revealed that lignicolous freshwater fungi constitute a highly diverse taxonomic group with a substantial population. To date, more than 3,870 species of freshwater fungi have been documented from various substrates and geographical locations (Luo et al., 2019; Dong et al., 2020; Shen et al., 2022; Calabon et al., 2023a, b). Lignicolous freshwater fungi are complex assemblages of mostly filamentous fungi, single-celled chytrids, and yeasts (Tsui et al., 2016). Freshwater fungi belong to Ascomycota, Basidiomycota, Chytridiomycota, and Rozellomycota; in class levels within the Ascomycota, most species are accommodated in Dothideomycetes and Sordariomycetes (Calabon et al., 2022).

*Chaetopsina* (family Nectriaceae) was established by Rambelli (1956), with the type species *Chaetopsina fulva*, which was isolated from decaying leaves in northern Italy. *Chaetopsina* is a dematiaceous hyphomycete genus, the members of which occur on decaying wood, leaves, pine needle litter, and bark, as well as on ascomycetous stromata and soil (Seifert et al., 2011). Most *Chaetopsina* species are found in tropical and subtropical areas (Lechat and Fournier, 2019). *Chaetopsina* is initially characterized by reddish-brown setose conidiophores turning yellow in lactic acid, and hyaline, smooth, fusiform ameroconidia, and fertile regions situated terminally or along the axes of the setiform conidiophores (Samuels, 1985; Kirk and Sutton, 1985). Later, some species with dark brown conidiophores or lateral branches on the conidiophores also have been accommodated (Bakhit and Abdel-Aziz, 2021). According to Luo and Zhuang (2010), *Chaetopsinectria chaetopsinae* was distinct as the sexual morph of *C. fulva*. Lombard et al. (2015) and Rossman et al. (2016) subsequently recommended using *Chaetopsina* despite *Chaetopsinectria* in respect to the 1F = 1N policy, and hitherto, *Chaetopsinectria* was treated as a synonym of *Chaetopsina*. The sexual morph of *Chaetopsina* is seen featuring superficial, non-stromatic, reddish brown to bright red, oval ascomata with an acute ostiolar apex, with walls composed of cells of *textura epidermoidea*, with paler papilla; unitunicate, clavate, short-stipitate asci containing 8-spored, fusiform, 0–1-septate, hyaline ascospores, and growing in association with upright conidiophores (Lechat and Fournier, 2020). So far, a total of 33 species epithets are listed under *Chaetopsina*, and four epithets are listed under *Chaetopsinectria* in Index Fungorum (https://indexfungorum.org/Names/Names.asp; accessed on 21 June 2024). However, eight previously described *Chaetopsina* epithets have been synonymized under other genera in Nectriaceae or *incertae sedis* (Index Fungorum, 2024), and the genus now embodies only 25 accepted species wherein molecular data of only 13 species are available (Lechat and Fournier, 2019; Bakhit and Abdel-Aziz, 2021). There are four species in *Chaetopsina* that have so far been recorded from freshwater habitats, including *Chaetopsina aquatica*, *C. fulva*, *Chaetopsina hongkongensis*, and *Chaetopsina polyblastia* (Sivichai et al., 2000; Ho et al., 2002; Luo et al., 2019; Bakhit and Abdel-Aziz, 2021). *C. fulva* and *C. polyblastia* were found on woody test blocks of *Xylia dolabriformis* in a freshwater stream of Khao Yai National Park, Thailand (Sivichai et al., 2000). *C. hongkongensis* was found on decaying submerged wood in Tai Po Kau Forest Stream, Hong Kong, China (Ho et al., 2002). *Chaetopsina beijingensis* (treated herein as a synonym of *C. fulva*) was isolated from decaying wood submerged in a freshwater stream in Yunnan, China (Luo et al., 2019). *C. aquatica* was collected on decaying submerged stems of *Phragmites australis* (Poaceae) in the River Nile, Sohag, Egypt (Bakhit and Abdel-Aziz, 2021).

*Parafuscosporella* (family Fuscosporellaceae), typified by *Parafuscosporella moniliformis*, was introduced by Yang et al. (2016). Yang et al. (2016) also introduced the new order Fuscosporellales to accommodate a new single family Fuscosporellaceae and to which six genera, viz., *Bactrodesmiastrum*, *Fuscosporella*, *Mucispora*, *Parafuscosporella*, *Plagiascoma*, and *Pseudoascotaiwania* were initially accommodated. *Parafuscosporella* is one of four new genera established by Yang et al. (2016) when the new order Fuscosporellales and the new family Fuscosporellaceae were established. The genus is characterized by spherical to cushion-shaped, black, gelatinous sporodochia, with a jelly-like cover, semi-macronematous, mononematous, compact, flexuous, simple or branched, mostly moniliform, globose to subglobose, ellipsoidal or clavate celled conidiophores, monoblastic, integrated, sometimes discrete, terminal, globose or subglobose, ellipsoidal or clavate conidiogenous cells and acrogenous, ellipsoidal to broadly obpyriform, smooth, dark brown to black conidia with a septum near the base, sometimes with a small protuberance and a pale brown basal cell (Boonyuen et al., 2016; Yang et al., 2016). Ten species are listed in *Parafuscosporella*, comprising *Parafuscosporella aquatica*, *Parafuscosporella ellipsoconidiogena*, *Parafuscosporella garethii*, *Parafuscosporella lignicola*, *Pa. moniliformis*, *Parafuscosporella mucosa*, *Parafuscosporella nilotica*, *Parafuscosporella obovata*, *Parafuscosporella pyriformis*, and *Parafuscosporella xishuangbannaensis* (Yang et al., 2016, 2020; Boonyuen et al., 2021; Boonmee et al., 2021; Wang et al., 2023; Li et al., 2023). Species of the genus have been reported from freshwater habitats in China and Thailand (Yang et al., 2016, 2020; Boonmee et al., 2021; Boonyuen et al., 2021). Goh et al. (2023) introduced two novel freshwater fungi from Taiwan, namely, *Vanakripa oblonga* and *Vanakripa taiwanensis*. Based on the morphological resemblance between *Parafuscosporella* and *Vanakripa*, Goh et al. (2023) treated *Parafuscosporella* as a synonym of *Vanakripa* and transferred all *Parafuscosporella* species to *Vanakripa*. In contrast, *Vanakripa chiangmaiensis* and *Vanakripa minutiellipsoidea* clustered with *Conioscypha* species were excluded from *Vanakripa* (Goh et al., 2023). Unfortunately, the type species of *Vanakripa*, *Vanakripa gigaspora*, lacks molecular data to clarify the phylogenetic placement. Hence, the congeneric status of *Parafuscosporella* and *Vanakripa* is questionable.

*Pleurotheciella* (family Pleurotheciaceae), typified by *Pleurotheciella rivularia*, was introduced by Réblová et al. (2012). To date, within the genus *Pleurotheciella*, only three species, viz., *Pleurotheciella erumpens*, *Pleurotheciella fusiformis*, and *Pl. rivularia*, are known for their sexual morphs, with non-stromatic perithecia, unitunicate asci, and hyaline to subhyaline, 1- or 3–5-septate ascospores, lacking a mucilaginous sheath. The asexual morph of the genus is a dactylaria-like hyphomycete, which is characterized by polyblastic, denticulate conidiogenesis, subhyaline conidiophores, and hyaline, ellipsoidal to ellipsoidal-fusiform, aseptate to multi-septate conidia (Luo et al., 2018). At present, 18 species are accommodated in *Pleurotheciella*, viz., *Pleurotheciella aquatica*, *Pleurotheciella centenaria*, *Pleurotheciella dimorphospora*, *Pl. erumpens*, *Pl. fusiformis*, *Pleurotheciella ganzhouensis*, *Pleurotheciella guttulata*, *Pleurotheciella irregularis*, *Pleurotheciella krabiensis*, *Pleurotheciella lunata*, *Pleurotheciella nilotica*, *Pl. rivularia*, *Pleurotheciella saprophytica*, *Pleurotheciella submersa*, *Pleurotheciella sympodia*, *Pleurotheciella tropica*, *Pleurotheciella uniseptata*, and *Pleurotheciella verrucosa*, and all accepted species of the genus have been reported for their asexual morphs (Hyde et al., 2018; Luo et al., 2018; Abdel-Aziz et al., 2020; Réblová et al., 2020; Shi et al., 2021; Boonmee et al., 2021; He et al., 2024). The genus has been so far represented from freshwater habitats in China and Thailand, except for *Pl. dimorphospora*, which is the only species described from terrestrial habitats in China (Boonmee et al., 2021).

In this study, we introduce three new species, *Chaetopsina yunnanensis*, *Parafuscosporella hunanensis*, and *Pleurotheciella yunnanensis*, based on morphology and phylogenetic studies, from freshwater habitats in China. In addition, *C. beijingensis* is formally synonymized under *C. fulva*, and the congeneric status of *Parafuscosporella* and *Vanakripa* is discussed.

## 2 Materials and methods

### 2.1 Sample collection, morphological studies, and isolation

Submerged decaying wood and branches were collected from a freshwater stream and lakes in Hunan and Yunnan provinces, China, from July to September 2022 (wet season). Detailed environmental parameters (e.g., pH, temperature, and dissolved oxygen) were recorded for each sample collected from lakes. Fresh specimens were brought to the laboratory in Ziploc bags and studied following the methods described by Luo et al. (2018). The samples were incubated in high-density plastic boxes lined with moisturized absorbent paper at room temperature for 1 week. Macro-morphological characters of the fungi on the host surface were observed using an Optec SZ760 compound stereomicroscope. Temporarily prepared microscope slides were placed under a Nikon ECLIPSE 80i compound microscope fitted with a Nikon DS-Ri2 digital camera for observation and micro-morphological photography. The morphologies of colonies on the substrates were photographed using a Nikon SMZ1000 stereo zoom microscope. Microscopic structures were measured using the Tarosoft^®^ Image Frame Work program, and the photographic plates were processed using Adobe Photoshop CS6 version 10.0 software (Adobe Systems, San Jose, CA, USA).

Single spore isolation was performed following the method described by Luo et al. (2018). The germinated conidia were aseptically transferred to fresh potato dextrose agar (PDA) plates and incubated at room temperature. The specimens were dried under natural light, wrapped in absorbent paper, and placed in a Ziploc bag with mothballs. Herbarium specimens were deposited in the Herbarium of Cryptogams, Kunming Institute of Botany Academia Sinica (KUN-HKAS), Kunming, China. The cultures were deposited in Kunming Institute of Botany, Chinese Academy of Sciences (KUNCC), Kunming, Yunnan, China. The novel species were registered in the Index Fungorum repository (https://indexfungorum.org/Names/IndexFungorumRegisterName.asp; accessed on 27 September 2024).

### 2.2 DNA extraction, PCR amplification, and sequencing

Fresh mycelia were scraped from colonies grown on PDA medium. DNA extraction was carried out using a DNA extraction kit (TOLOBIO Plant Genomic DNA Extraction Kit, Tsingke Company, Beijing, China) following the manufacturer’s instructions. PCR amplification was performed using primer pairs LR0R/LR5 (Vilgalys and Hester, 1990) for the nuclear ribosomal large subunit 28S rDNA gene (LSU), NS1/NS4 (White et al., 1990) for the nuclear ribosomal small subunit 18S rDNA gene (SSU), ITS5/ITS4 (White et al., 1990) for the internal transcribed spacer rDNA region (ITS), and fRPB2-5F/fRPB2-7cR (Liu et al., 1999; Rehner and Buckley, 2005) for the RNA polymerase second largest subunit (*RPB2*). The PCR amplification was carried out in a 25-μL reaction volume containing 12.5 μL of 2× Power Taq PCR Master Mix, 1 μL of each forward and reward primer (10 μM), 1 μL of genomic DNA template (30–50 ng/μL), and 9.5 μL sterilized double-distilled water. Amplifications were carried out using the BioTeke GT9612 thermocycler (Tsingke Company, Beijing, China). The PCR amplification conditions for ITS, LSU, and SSU consisted of initial denaturation at 98°C for 3 minutes, followed by 35 cycles of denaturation at 98°C for 20 seconds, annealing at 53°C for 10 seconds, an extension at 72°C for 20 seconds, and a final extension at 72°C for 5 minutes. The PCR amplification conditions for *RPB2* consisted of initial denaturation at 95°C for 5 minutes, followed by 40 cycles of denaturation at 95°C for 1 minute, annealing at 52°C for 2 minutes, an extension at 72°C for 90 seconds, and a final extension at 72°C for 10 minutes. The quality of PCR products was checked using 1% agarose gel electrophoresis, and distinct bands were visualized in the gel documentation system (Compact Desktop UV Transilluminator analyzer GL-3120). The PCR products were purified, and Sanger sequences were obtained by Tsingke Company, Beijing, China.

### 2.3 Sequence assembly, alignment, and phylogenetic analyses

The newly generated sequences were subjected to the nucleotide BLAST search via the NCBI (https://blast.ncbi.nlm.nih.gov/Blast.cgi; accessed on 1 May 2024) to search the closely related taxa and confirm the correctness of the sequences. The sequence datasets were obtained by compiling the closely related taxa of the novel taxa retrieved from GenBank based on nucleotide BLAST searches and recent publications (Luo et al., 2018; Lechat and Fournier, 2020; Bakhit and Abdel-Aziz, 2021; Boonmee et al., 2021; Shi et al., 2021; Li et al., 2023; He et al., 2024). Outgroups were selected based on recently published data (Bakhit and Abdel-Aziz, 2021; Shi et al., 2021; Li et al., 2023) (**Tables 1**–**3**). Multiple sequence alignments were aligned with MAFFT v.7 (http://mafft.cbrc.jp/alignment/server/index.html; accessed on 10 May 2024) (Katoh et al., 2019) and automatically trimmed using TrimAl (http://phylemon.bioinfo.cipf.es/utilities.html; accessed on 10 May 2024) (Capella-Gutiérrez et al., 2009). A combined sequence dataset was obtained using SquenceMatrix v.1.7.8 (Vaidya et al., 2011). Phylogenetic relationships of the new taxa were performed based on maximum likelihood (ML) and Bayesian inference (BI) analyses.

**Table 1:** Taxon names, strain numbers, and GenBank accession numbers of the ITS and LSU sequences used in the phylogenetic analyses of *Chaetopsina*.

Taxon name	Voucher/culture	GenBank accession numbers	
		ITS	LSU
*Calonectria parvispora*	CBS 111465	MT359775	MT359535
*C. parvispora*	CMW 30981	MT359774	MT359534
***Chaetopsina acutispora***	**CBS 667.92**	**MH862382**	**MH874045**
***Chaetopsina aquatica***	**SUMCC H-18001**	**MW633072**	**MW633073**
***Chaetopsina aurantisalinicola***	**MFLU 18-0566**	**NR_168213**	**NG_068297**
*C. aurantisalinicola*	MFLUCC 17-0414	MN047103	MN017868
***Chaetopsina eucalypti***	**CPC 32857**	**MH327799**	**MH327835**
*Chaetopsina fulva*	CBS 138004	KJ869159	KJ869216
*C. fulva*	MFLU 18-2327	MK828667	MK828234
***C. fulva***	**CBS 142.56**	**KM231772**	**NG_070573**
*C. fulva*	FMR 13129	KY853432	KY853492
*C. fulva*	HMAS 188462	GU075861	GU075867
***Chaetopsina gautengina***	**CPC 34896**	**NR_170049**	**NG_073870**
***Chaetopsina penicillata***	**CBS 608.92**	**NR_154780**	**NG_058781**
*C. penicillata*	KUNCC22-12664	OP985128	OP985134
***Chaetopsina pini***	**CPC 21622**	**KF777144**	**KF777200**
***Chaetopsina pinicola***	**CPC 21819**	**NR_137823**	**KF777201**
***Chaetopsina pnagiana***	**BRFM 3055**	**NR_175643**	**NG_088111**
***Chaetopsina saulensis***	**CLLG 18029**	**MN017104**	**MN017106**
***Chaetopsina yunnanensis***	**KUNCC23-12940**	**OQ860234**	**PP151255**
*C. yunnanensis*	KUNCC23-13014	OQ860233	PP151256
***Graphium carbonarium***	**CBS 123610**	**MH863310**	**MH874834**
***Graphium jumulu***	**CBS 139898**	**NR_137980**	**NG_069278**
*Volutella gilva*	CBS 128258	MH864864	MH876309
***Volutella leucaenae***	**MFLUCC 17-2620**	**NR_189395**	**NG_241997**
***Volutella salvadorae***	**CBS 147070**	**NR_173060**	**NG_076746**
***Volutella thailandensis***	**MFLUCC 16-0366**	**NR_169676**	**MH376742**

The newly generated sequences are indicated in red, while the type strains are in black bold font. “–” indicates unavailable sequences.

ITS, internal transcribed spacer; LSU, large subunit.

**Table 2:** Taxon names, strain numbers, and GenBank accession numbers of the ITS, LSU, and SSU and sequences used in the phylogenetic analyses of *Parafuscosporella*.

Taxon name	Voucher/culture	GenBank accession number		
		ITS	LSU	SSU
***Fuscosporella aquatica***	**MFLUCC 16–0859**	**NR_156398**	**NG_059853**	**NG_062433**
***Fuscosporella pyriformis***	**MFLUCC 16–0570**	**NR_152555**	**KX550896**	**NG_061248**
*Parafuscosporella aquatica*	KUMCC 19–0211	NR_173178	MN512343	–
***Parafuscosporella ellipsoconidiogena***	**TBRC 15503**	**OK044749**	**OK044741**	**OK054346**
*Pa. ellipsoconidiogena*	TBRC 15504	OK044750	OK044742	OK054347
***Parafuscosporella garethii***	**BCC79986**	**OK135602**	**KX958430**	**KX958428**
*Pa. garethii*	BCC79987	OK135603	KX958431	KX958429
***Parafuscosporella lignicola***	**MFLUCC 23–0047**	**OQ917244**	**OQ875867**	**OQ917245**
*Pa. lignicola*	MFLUCC 23–0048	OQ925403	OQ875868	OQ925402
***Parafuscosporella moniliformis***	**MFLUCC 15**–**0626**	**NR_152557**	**KX550895**	**NG_063614**
***Parafuscosporella mucosa***	**MFLUCC 16**–**0571**	**MG388214**	**NG_059855**	**NG_063663**
***Parafuscosporella hunanensis***	**KUNCC23**–**13574**	**OR230704**	**PP744555**	**PP744557**
*Pa. hunanensis*	KUNCC24–17774	PP744554	PP744556	PP744558
***Parafuscosporella nilotica***	**CBS H22128**	**MN921198**	–	**MN921199**
***Parafuscosporella obovata***	**TBRC 15505**	**OK044751**	**OK044743**	**OK054348**
*Parafuscosporella pyriformis*	MFLUCC 18–1400	MN513030	MN512339	–
*Pa. pyriformis*	KUMCC 19–0008	MN513031	MN512340	–
***Parafuscosporella xishuangbannaensis***	**IFRDCC 3133**	**ON540716**	**ON540747**	–
***Vanakripa oblonga***	**BCRC FU31423**	**MT452512**	**OQ079570**	**OQ079568**
***Vanakripa taiwanensis***	**BCRC FU31428**	**MT452513**	**OQ079569**	**OQ079567**

The newly generated sequences are indicated in red, while the type strains are in black bold font. “–” indicates unavailable sequences.

ITS, internal transcribed spacer; LSU, large subunit; SSU, small subunit.

**Table 3:** Taxon names, strain numbers, and GenBank accession numbers of the ITS, LSU, SSU, and *RPB2* sequences used in the phylogenetic analyses of *Pleurotheciella*.

Taxon name	Voucher/culture	GenBank accession numbers			
		ITS	LSU	SSU	*RPB2*
*Pleurotheciella aquatica*	MFLU 17-0911	NR_160591	NG_066193	–	–
***Pl. aquatica***	**MFLUCC 17-0464**	**MF399236**	**MF399253**	**MF399220**	**MF401405**
***Pleurotheciella centenaria***	**DAOM 229631**	**NR_111709**	**NG_060098**	**NG_064996**	**JQ429265**
***Pleurotheciella dimorphospora***	**KUMCC 20-0185**	**NR_175737**	**NG_081519**	**NG_078760**	**–**
***Pl. dimorphospora***	**KIB049/MFLU 20-0138**	**MW981446**	**MW981444**	**MW981454**	**MZ509665**
***Pleurotheciella erumpens***	**CBS 142447**	**NR_170010**	**MN699435**	**NG_070323**	**MN704311**
*Pleurotheciella fusiformis*	IFRD500 014	MT555417	MT559121	MT555733	–
***Pleurotheciella ganzhouensis***	**JAUCC6079**	**OR853417**	**OR853422**	**OR853426**	**PP078759**
*Pl. ganzhouensis*	JAUCC6678	PP800192	PP800214	PP801261	PP816289
*Pleurotheciella guttulata*	**KUMCC 15-0442**	**MF399239**	**MF399256**	**MF399222**	**MF401408**
***Pl. guttulata***	**KUMCC 15-0296**	**MF399240**	**MF399257**	**MF399223**	**MF401409**
***Pleurotheciella irregularis***	**JAUCC6080**	**OR853418**	**OR853423**	**PP801258**	**PP816286**
*Pl. irregularis*	JAUCC6679	PP800193	–	PP801262	–
***Pleurotheciella krabiensis***	**MFLUCC 16-0852**	**MG837018**	**MG837013**	**MG837023**	**–**
*Pl. krabiensis*	MFLUCC 18-0856	MG837019	MG837014	MG837024	–
***Pleurotheciella lunata***	**S-426**	**MK878378**	**MK835847**	**MK834782**	**–**
***Pl. lunata***	**MFLUCC 17-0111**	**MF399238**	**MF399255**	**MF399221**	**MF401407**
***Pleurotheciella rivularia***	**CBS 125238**	**NR_111711**	**NG_057950**	**NG_061124**	**JQ429263**
***Pleurotheciella saprophytica***	**MFLUCC 16-1251**	**MF399241**	**MF399258**	**MF399224**	**MF401410**
***Pleurotheciella submersa***	**MFLUCC 17-1709**	**MF399243**	**MF399260**	**MF399226**	**MF401412**
*Pl. submersa*	DLUCC 0739	MF399242	MF399259	MF399225	MF401411
*Pleurotheciella sympodia*	MFLUCC 18-0983	MT555419	MT555425	MT555734	–
*Pl. sympodia*	MFLUCC 18-0658	MT555418	MT559086	MT559094	–
*Pl. sympodia*	KUMCC 19-0213	MT555420	MT555426	–	–
*Pleurotheciella tropica*	MFLU 18-0141	MG837020	MG837015	MG837025	–
*Pleurotheciella uniseptata*	S-936	MK878377	MK835846	MK834781	MN194025
***Pleurotheciella verrucosa***	**JAUCC6076**	**OR853414**	**OR853419**	**OR853424**	**PP078756**
*Pl. verrucosa*	JAUCC6675	PP800189	PP800211	PP801259	PP816287
*Pl. verrucosa*	JAUCC6078	OR853416	OR853421	PP801257	PP078758
*Pl. verrucosa*	JAUCC6677	PP800191	PP800213	–	–
***Pleurotheciella yunnanensis***	**KUNCC23-13328**	OR234682	PP095383	PP095382	PP131261
*Pl. yunnanensis*	KUNCC23-13682	PP095384	PP095381	PP095385	PP131262
*Rhexoacrodictys erecta*	HSAUPmyr4622	KU999964	KX033556	KX033526	–
*Rhexoacrodictys fimicola*	HMAS 47737	KU999960	KX033553	KX033522	–

The newly generated sequences are indicated in red, while the type strains are in black bold font. “–” indicates unavailable sequences.

BCC, BIOTEC Culture Collection, Pathum Thani, Thailand; BCRC, Bioresource Collection and Research Centre, Food Industry Research and Development Institute, Hsinchu, Taiwan; BRFM, Biological Resource Center CIRM-CF (International Center of Microbial Resources, Marseille, France; CBS, Culture Collection of the Westerdijk Fungal Biodiversity Institute, Utrecht, Netherlands; CMW, Culture collection of the Forestry and Agricultural Biotechnology Institute (FABI), University of Pretoria, Pretoria, South Africa; CLLG, Collection of Christian Lechat at French GuIana deposited in LIP herbarium (university of Lille); CPC, Culture Collection of Pedro Crous, Netherlands; FMR, Facultat de Medicina i Ciencies de la Salut, Reus, Spain; DAOM, Canadian Collection of Fungal Cultures, Agriculture and Agri-Food Canada, Ottawa, Canada; DLUCC/HMAS, Mycological Herbarium, Institute of Microbiology, Chinese Academy of Sciences, Beijing, China; HSAUP, Herbarium of Department of Plant Pathology, Shandong Agricultural University, Taian, Shandong, China; IFRD/IFRDCC, Research Institute of Resources Insects, China Academy of Forestry; JAUCC, Jiangxi Agricultural University Culture Collection; KIB, Collection of Rungtiwa Phookamsak at Kunming Institute of botany, Chinese Academy of Sciences; KUMCC/KUNCC, Kunming Institute of Botany, Chinese Academy of Sciences Culture Collection, Kunming, Yunnan, China; MFLU, the herbarium of Mae Fah Luang University, Chiang Rai, Thailand; MFLUCC, Mae Fah Luang University Culture Collection, Chiang Rai, Thailand; S, Collection of Hongyan Su; SUMCC, Sohag University microbial culture collection, Egypt; TBRC, Thailand Bioresource Research Center; ITS, internal transcribed spacer; LSU, large subunit; SSU, small subunit.

ML analysis was performed by RAxML-HPC2 v.8.2.12 on the XSEDE (8.2.12) tool via the CIPRES Science Gateway (http://www.phylo.org/portal2; accessed on 25 May 2024) (Stamatakis, 2006; Miller et al., 2015) following the default setting but adjusted by setting 1,000 bootstrap replications and GTRGAMMA model of nucleotide substitution. The evolutionary model of nucleotide substitution for the BI analyses was performed independently for each locus using MrModeltest v 2.3 (Nylander, 2008). GTR+I+G was selected as the best-fit model for ITS, LSU, SSU, and *RPB2* datasets in all analyses under the Akaike information criterion (AIC). Markov Chain Monte Carlo (MCMC) sampling was computed to estimate Bayesian posterior probabilities (BYPP) in MrBayes v.3.2.7 (Ronquist et al., 2012). Two parallel runs with six simultaneous Markov chains were run for 1,000,000 generations but stopped automatically when the critical value for the topological convergence diagnostic reached 0.01. Trees were sampled every 200th generation. The first 10% of the total trees were set as burn-in and were discarded. The remaining trees were used to calculate posterior probabilities in the majority rule consensus tree.

Phylograms were visualized using FigTree v1.4.4 (Rambaut, 2018) and rearranged in Adobe Photoshop CS6 software (Adobe Systems, USA). The new sequences were deposited in GenBank (**Tables 1** **–** **3**), and the final alignment and phylogenetic tree were registered in TreeBASE under the submission IDs: 31916 (*C. yunnanensis*), 31910 (*Pa. hunanensis*) and 31135 (*Pl. yunnanensis*) (http://www.treebase.org/ accessed on 25 December 2024).

## 3 Results

### 3.1 Phylogenetic analyses

#### Analysis 1

The concatenated LSU and ITS sequence dataset comprises 27 representative taxa in *Calonectria*, *Chaetopsina*, and *Volutella* with *Graphium carbonarium* (CBS 123610) and *Graphium jumulu* (CBS 139898) as the outgroup taxa (**Table 1**). The concatenated sequence matrix comprises 1,420 characters, including gaps (LSU, 828 bp; ITS, 588 bp). BI and ML analyses of the combined dataset were performed to determine the placement of our new taxon and infer relationships at the intrageneric level as well as resolve the phylogenetic relationships of the core genera in Nectriaceae. The phylogenetic trees obtained from BI and ML analyses resulted in trees with largely similar topologies. A phylogenetic investigation based on ML analysis was carried out with the best RAxML tree with a final likelihood value of −5,889.450811. The matrix had 385 distinct alignment patterns, with 6.19% undetermined characters or gaps. Estimated base frequencies were as follows: A = 0.238371, C = 0.250160, G = 0.286306, T = 0.225163, with substitution rates AC = 1.768750, AG = 2.223229, AT = 2.277765, CG = 1.698772, CT = 7.040906, GT = 1.000000; gamma distribution shape parameter α = 0.526092. The final average standard deviation of split frequencies at the end of total MCMC generations was calculated as 0.009709 in BI analysis.

**Figure 1:**
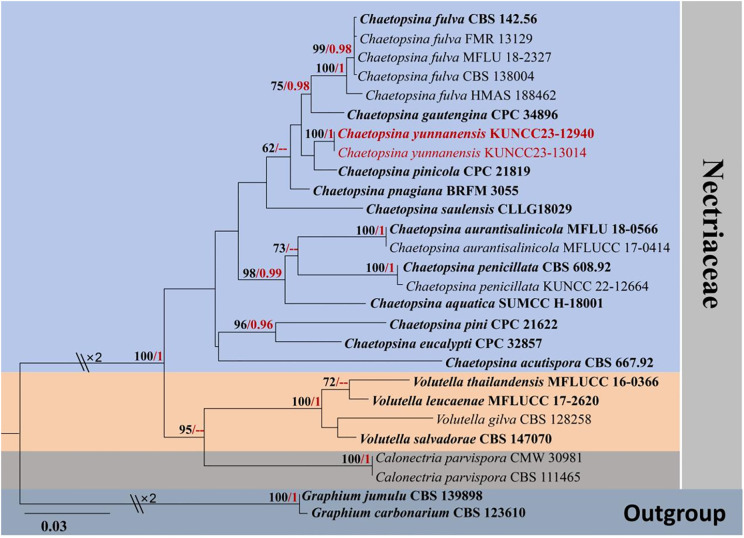
RAxML phylogenetic tree of a concatenated LSU and ITS sequence dataset. The BI and ML support values equal to or greater than 0.90 BYPP and 60% MLBS are shown as “MLBS/BYPP” at the nodes. The tree is rooted to *Graphium carbonarium* (CBS 123610) and *Graphium jumulu* (CBS 139898). Type strains are in bold, and newly generated strains are in red. LSU, large subunit; ITS, internal transcribed spacer; BI, Bayesian inference; ML, maximum likelihood.

Molecular analysis of a concatenated LSU and ITS sequence dataset (**Figure 1**) demonstrated that the phylogenetic relationship of the representative genera, *Calonectria*, *Chaetopsina*, and *Volutella*, is not well-resolved. The genera *Calonectria* and *Volutella* formed distinct clades with well-supported value in ML analyses but low-supported value in BI analysis. *Chaetopsina* formed a distinct clade with *Calonectria* and *Volutella* with low support. The interspecific status of many *Chaetopsina* species is not well-resolved in the present study, including *Chaetopsina acutispora*, *Chaetopsina pinicola*, *Chaetopsina pnagiana*, and *Chaetopsina saulensis*. Two new strains (KUNCC23-12940 and KUNCC23-13014) formed a robust subclade [100% maximum likelihood bootstrap support (MLBS) and 1.00 Bayesian posterior probabilities (BYPP)] and clustered with *C. pinicola* (CPC 21819) with low-supported values. *C. beijingensis* (CBS 138004) shared the same branch with the type (CBS 142.56) and representative strains of *C. fulva* (FMR 13129 and MFLU 18-2327). In contrast, *C. fulva* (HMAS 188462) remained distinct from other strains.

#### Analysis 2

**Figure 2:**
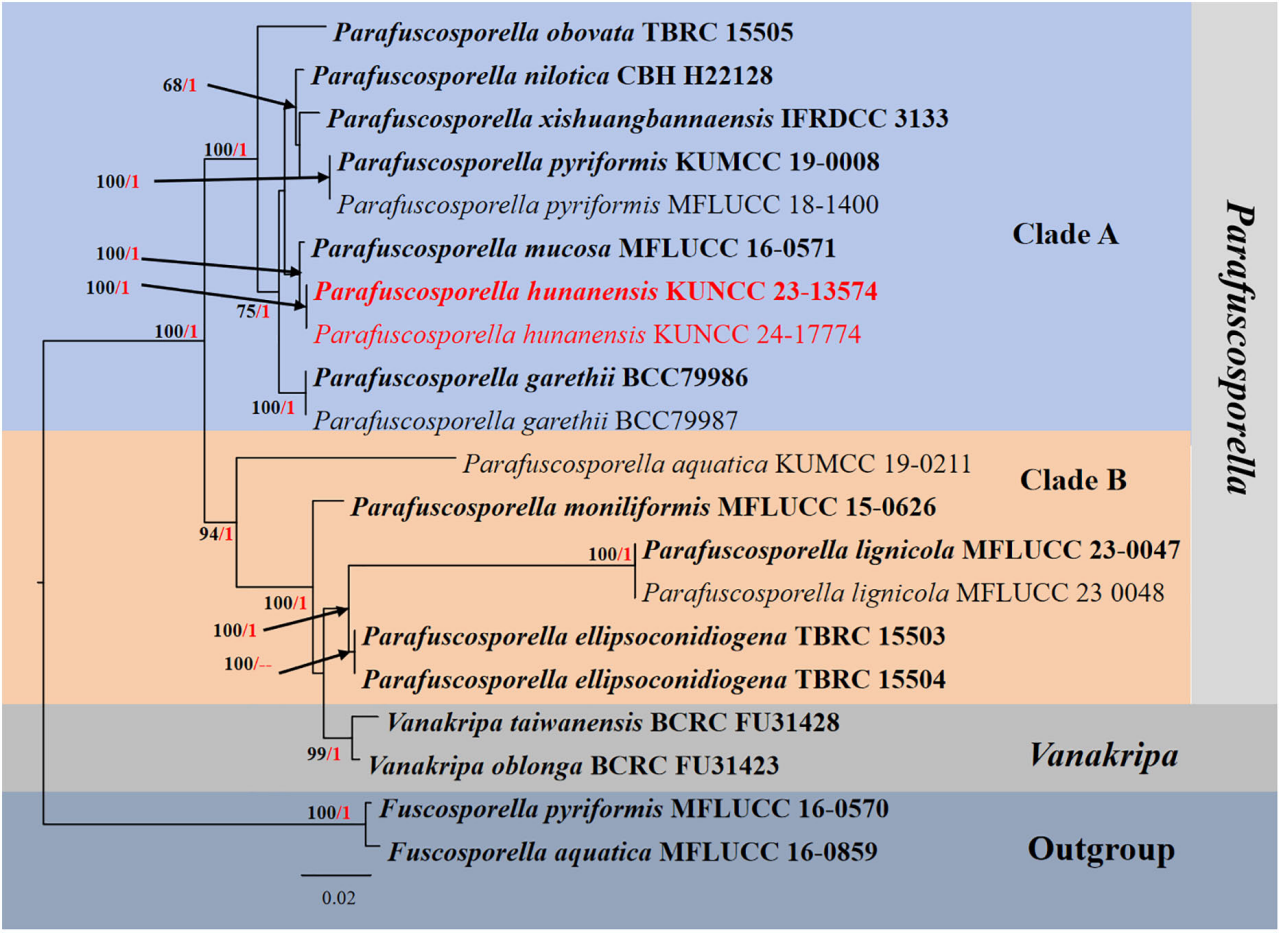
RAxML phylogenetic tree of a concatenated ITS, LSU, and SSU sequence datasets. The BI and ML support values equal to or greater than 0.90 BYPP and 60% MLBS are shown as “MLBS/BYPP” at the nodes. The tree is rooted to *Fuscosporella aquatica* (MFLUCC 16–0859) and *Fuscosporella pyriformis* (MFLUCC 16–0570). Type strains are in bold, and newly generated strains are in red. ITS, internal transcribed spacer; LSU, large subunit; SSU, small subunit; BI, Bayesian inference; ML, maximum likelihood.

The phylogenetic analyses of concatenated ITS, LSU, and SSU sequence data were conducted to demonstrate the phylogenetic relationships of the new isolates with other species in *Parafuscosporella*. Twenty strains were included in the combined dataset (**Table 2**), which comprised 2,340 characters (ITS, 535 bp; LSU, 829 bp; SSU, 976 bp) after alignment (including gaps). The best RAxML tree with a final likelihood value of −6,818.339932 is presented (**Figure 2**). RAxML analysis yielded 415 distinct alignment patterns and 16.25% of undetermined characters or gaps. Estimated base frequencies were as follows: A = 0.231018, C = 0.253572, G = 0.287376, T = 0.228033, with substitution rates AC = 1.005383, AG = 1.978020, AT = 1.365847, CG = 0.802886, CT = 4.380984, GT = 1.000000; gamma distribution shape parameter alpha = 0.765977. The final average standard deviation of split frequencies at the end of total MCMC generations for BI analysis was 0.009867.

Phylogenetic analyses retrieved from ML and BI analyses were not significantly different and showed similar topologies. Phylogenetic analyses demonstrated that *Parafuscosporella* formed the well-resolved clade and separated into two subclades: clade A comprises *Pa. garethii*, *Pa. hunanensis*, *Pa. mucosa*, *Pa. nilotica*, *Pa. obovata*, *Pa. pyriformis*, and *Pa. xishuangbannaensis*, whereas clade B comprises *Pa. aquatica*, *Pa. ellipsoconidiogena*, *Pa. lignicola*, and *Pa*. *moniliformis*. Two *Vanakripa* species, *V. oblonga* and *V. taiwanensis*, also formed a high-support subclade closely related to *Pa. ellipsoconidiogena* and *Pa. lignicola*. The interspecific status of most *Parafuscosporella* is well-clarified, except for *Pa. xishuangbannaensis*, in the present study. The two strains (KUNCC23-13574 and KUNCC24-17774) of *Pa. hunanensis* sp. nov. clustered with *Pa. mucosa* in subclade A with 100% MLBS and 1.00 BYPP support values.

#### Analysis 3

**Figure 3:**
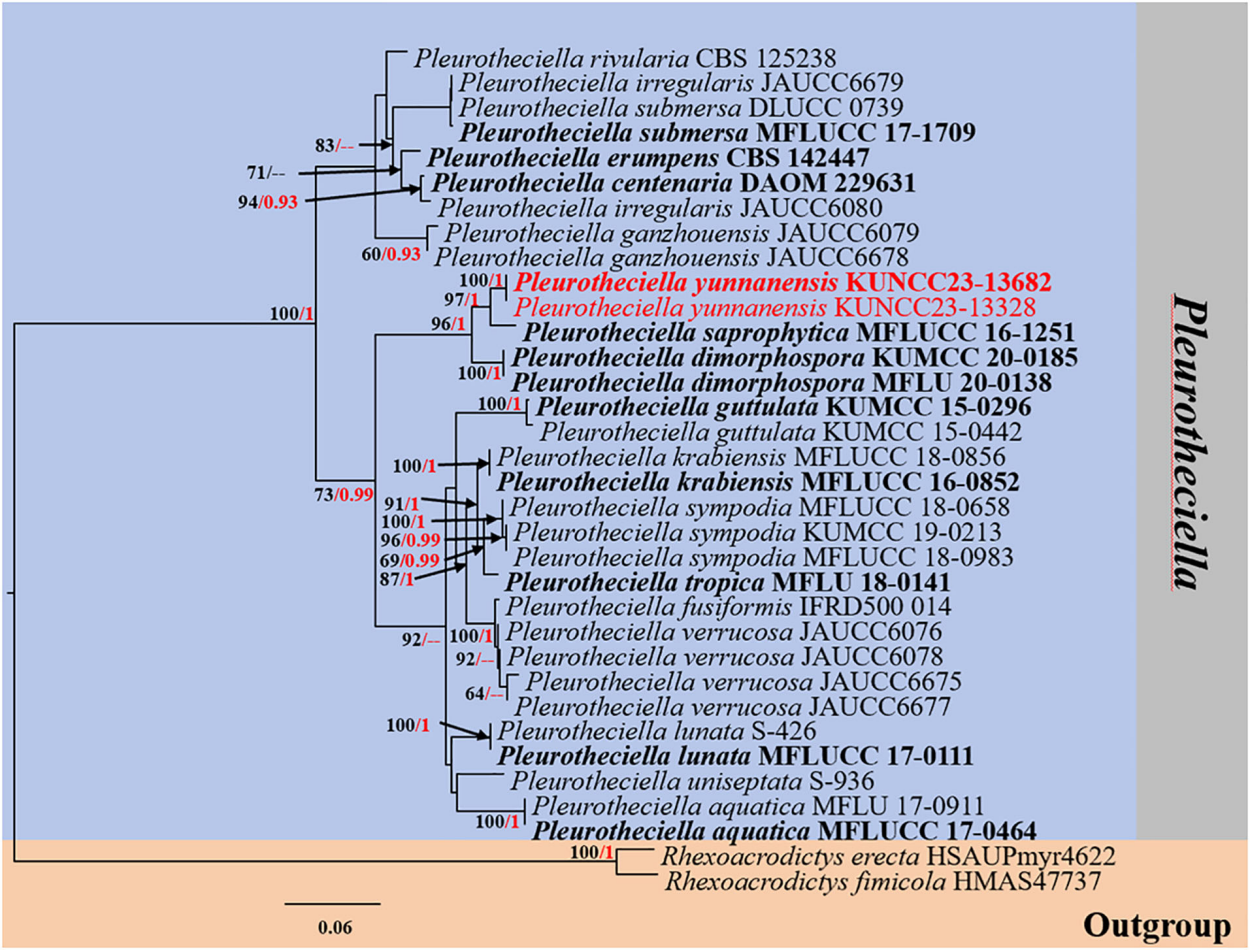
RAxML phylogenetic tree of a concatenated ITS, LSU, SSU, and *RPB2* sequence dataset. The BI and ML support values equal to or greater than 0.90 BYPP and 60% MLBS are shown as “MLBS/BYPP” at the nodes. The tree is rooted to *Rhexoacrodictys erecta* (HSAUPmyr4622) and *Rhexoacrodictys fimicola* (HMAS 47737). Type strains are in bold, and newly generated strains are in red. ITS, internal transcribed spacer; LSU, large subunit; SSU, small subunit; BI, Bayesian inference; ML, maximum likelihood.

The phylogenetic analyses of concatenated ITS, LSU, SSU, and *RPB2* sequence data were conducted to demonstrate the relationship between the new species and other known species in *Pleurotheciella*. Thirty-four strains were included in the combined dataset (**Table 3**), which comprised 2,919 characters (ITS, 564 bp; LSU, 825 bp; SSU, 682 bp; *RRB2*, 848 bp) after alignment (including gaps). *Rhexoacrodictys erecta* (HSAUPmyr4622) and *Rhexoacrodictys fimicola* (HMAS 47737) were selected as the outgroup taxa. The best RAxML tree with a final likelihood value of −10,754.565198 was selected to represent the phylogenetic affinity of the novel species with closely related species (**Figure 3**). RAxML analysis yielded 677 distinct alignment patterns and 18.84% of undetermined characters or gaps. Estimated base frequencies were as follows: A = 0.234132, C = 0.258625, G = 0.287873, T = 0.219371, with substitution rates AC = 1.285686, AG = 3.496823, AT = 1.635783, CG = 0.882608, CT = 9.634626, GT = 1.000000; gamma distribution shape parameter alpha = 0.461774. The final average standard deviation of split frequencies at the end of total MCMC generations for BI analysis was 0.009778.

Phylogenetic analyses retrieved from ML and BI analyses were not significantly different and showed similar topologies. Phylogenetic affinities of most *Pleurotheciella* species are well-resolved (up to 70% MLBS and 0.90 BYPP support values) in the present study, except for *Pl. uniseptata*. However, the species always formed a stable subclade with *Pl. aquatica*. Two strains (KUNCC23-13328 and KUNCC23-13682) of the new species, *Pl. yunnanensis*, formed a distinct subclade (100% MLBS/1.00 BYPP), sister to *Pl. saprophytica* (MFLUCC 16-1251) with significant support (96% MLBS/1.00 BYPP) and clustered with *Pl. dimorphospora* (KUMCC 20-0185) with significant support (96% MLBS/1.00 BYPP).

### 3.2 Taxonomy

**Class** Sordariomycetes O.E. Erikss. & Winka

**Subclass** Hypocreomycetidae O.E. Erikss. & Winka

**Order** Hypocreales Lindau

**Family** Nectriaceae Tul. & C. Tul.

**Genus** *Chaetopsina* Rambelli

***Chaetopsina fulva*** Atti Accad. Sci. Ist. Bologna, Cl. Sci. Fis., Rendiconti 11: 5 (1956). **Amend**. L. Li, Phookamsak & Bhat

Index Fungorum number: IF 294735

= *C. beijingensis* Crous & Y. Zhang ter, in Crous et al., Persoonia 32: 267 (2014)

Typification: Italy, on fallen leaves (needle) of *Cedrus deodara* (Pinaceae), Feb 1956, A. Rambelli, IMI 62199 (type of *C. fulva*), ex-type culture, CBS 142.56; China, Beijing, Fragrant Hill, N39°59′18.4″ E116°11′25″, on needles of *Pinus tabulaeformis* (Pinaceae), 1 Sep 2013, P.W. Crous & Y. Zhang, CBS H-21718 (holotype of *C. beijingensis*), CPC 23629 = CBS 138004.

*Saprobic* on Pinaceae and various plant hosts as well as soil. Sexual morph: Referred to *Chaetopsinectria chaetopsinae* (≡ *Nectria chaetopsinae* Samuels; Lombard et al., 2015; Rossman et al., 2016). Asexual morph: *Conidiophores* 180–300 × 7.5–10 µm, macronematous, simple, sparse, erect, setiform, often swollen, bulbous at the base (up to 15–20 µm), tapering toward acutely rounded apex, straight or slightly curved, mostly flexuous, yellow-brown to red-brown, less pigmented at the base, turning red-brown in 3% KOH and yellow in lactic acid, unbranched, smooth to verruculose, up to 13-septate (12–16-septate as in *C. beijingensis*), thick-walled (2 µm diam.), sterile or occasionally fertile; fertile region situated below the middle of the main axis or higher, occasionally terminal, comprising an irregularly branched, densely aggregated, hyaline, conidiogenous apparatus. *Conidiogenous cells* 6–12(–20) × 3.5–5 µm monophialidic, discrete, hyaline, ampulliform to lageniform, smooth, irregularly branched, periclinal thickening visible, with minute collarettes. *Conidia* 8–12 × 2 µm, solitary, hyaline, subcylindrical to cylindrical, with rounded ends, aseptate, smooth, with guttules, rarely with flattened hilum (adopted from Ellis, 1971; Kirk and Sutton, 1985; Samuels, 1985; Luo and Zhuang, 2010; Crous et al., 2014; Perera et al., 2023).

*Known habitats and host*: On needle of *C. deodara* and fallen leaves of *Laurus nobilis*, *Quercus*, and *Carpinus*, needles of *P. tabulaeformis* (as *C. beijingensis*), woody test blocks of *X. dolabriformis*, dead leaves, decaying wood and soil (Rambelli, 1956; Ellis, 1971; Sivichai et al., 2000; Luo and Zhuang, 2010; Crous et al., 2014; Arias et al., 2015; Hernández-Restrepo et al., 2017; Luo et al., 2019; Vu et al., 2019; Perera et al., 2023).

*Known distribution*: Canada, China, Italy, Spain, and Thailand (Rambelli, 1956; Ellis, 1971; Sivichai et al., 2000; Luo and Zhuang, 2010; Crous et al., 2014; Arias et al., 2015; Hernández-Restrepo et al., 2017; Luo et al., 2019; Vu et al., 2019; Perera et al., 2023).

*Notes*: *C. fulva* was designated as the type species of *Chaetopsina* when Rambelli (1956) introduced the genus. The species was poorly studied to begin with, and molecular data of the type specimen were obtained by Vu et al. (2019), who also confirmed its phylogenetic affinity in the Nectriaceae. The species, originally isolated from a needle of *C. deodara* and fallen leaves of *L. nobilis*, *Quercus*, and *Carpinus* in northern Italy, produces pigmented, erect, septate, setiform conidiophores that are apically sterile (Rambelli, 1956; Kirk and Sutton, 1985; Bakhit and Abdel-Aziz, 2021; Index Fungorum, 2024). Ellis (1971) provided a detailed morphological description of *C. fulva*, which produces macronematous, up to 280 × 5–8 µm conidiophores, with bases often swollen to 15–20 µm. The conidiogenous cells (phialides) are 7–15 µm long, with a swollen base 3–4 µm wide and a phialidic neck approximately 1 µm thick. Conidia are 7–11 × 1 µm. The species were reported from dead fallen leaves and soil, with distribution noted in Canada and Italy. Kirk and Sutton (1985) re-circumscribed the species based on type studies and emended the generic circumscription incorporating only five described species, viz., *Chaetopsina catenulata*, *C. fulva*, *Chaetopsina penicillata*, *C. polyblastia*, and *Chaetopsina splendida*. Kirk and Sutton (1985) treated *Chaetopsina romantica* (= *Chaetopsis romantica*) as a synonym of *C. fulva* on the insistence of Rambelli (1987).

Luo and Zhuang (2010) introduced a sexual genus *Chaetopsinectria* to accommodate sexual morphs of *Chaetopsina*. Based on ITS and 28S rDNA sequence analyses, Luo and Zhuang (2010) mentioned that *C. fulva*, *C. chaetopsinae*, and *C. chaetopsinae-penicillatae* have a close relationship. Regarding the generic type, *C. fulva*, the common features include red-brown and setose conidiophores that turn yellow in lactic acid, hyaline phialides, and sienna-colored colonies on PDA; these morphological traits are shared by other *Chaetopsina* asexual morph of nectriaceous fungi and provide phenotypic information in defining this asexual genus (Samuels, 1985; Okada et al., 1997; Luo and Zhuang, 2010). However, Rossman et al. (2016) mentioned that *C. fulva* is the asexual morph of *C. chaetopsinae* (≡ *N. chaetopsinae*), and hence, these generic names were synonyms and further recommended to use *Chaetopsina* rather than *Chaetopsinectria* due to its prior establishment, following the 1F = 1N policy (McNeill et al., 2012).

Crous et al. (2014) introduced *C. beijingensis*, collected from decaying wood submerged in a freshwater stream, in Beijing, China, and further demonstrated the similarity of nucleotide pairwise (ITS and LSU) between *C. beijingensis* and *C. fulva* (HMAS 188462) with 98% similarity of ITS and 100% similarity of LSU. Morphologically, *C. beijingensis* resembles *C. fulva* but can be distinguished from the latter in having subcylindrical and slightly larger conidia [(11–)12–13(–14) × 2(–2.5) μm; Crous et al., 2014]. In contrast, *C. fulva* (described from the type’s slide) has cylindrical, 8–12 × 1.5 μm conidia (Kirk and Sutton, 1985). Vu et al. (2019) provided the molecular data from the type of *C. fulva* (CBS 142.56). Subsequently, Lechat and Fournier (2019) informally treated *C. beijingensis* as a synonym of *C. fulva*, in view of their morphological resemblance, phylogenetic evidence, and nucleotide pairwise similarities—99.6% similarity of ITS and 100% similarity of LSU sequences based on the nucleotide pairwise comparison of the type strains (CBS 142.56 vs. CBS 138004)—but did not taxonomically synonymize these taxa. Phylogenetically, *C. beijingensis* (CBS 138004) shares the same branch with the type strain of *C. fulva* and other representative strains with high support (100% MLBS/1.00 BYPP; **Figure 1**) in the present study. In accordance with Lechat and Fournier (2019) and our own observations, we formally synonymized *C. beijingensis* under *C. fulva* based on morphological indistinctiveness and phylogenetic support coupled with the conspecific in nucleotide polymorphism. Detailed description is amended to incorporate the morphological features of *C. beijingensis* and *C. fulva*.

***Chaetopsina yunnanensis*** L. Li, Bhat & Phookamsak, **sp. nov.**

**Figure 4:**
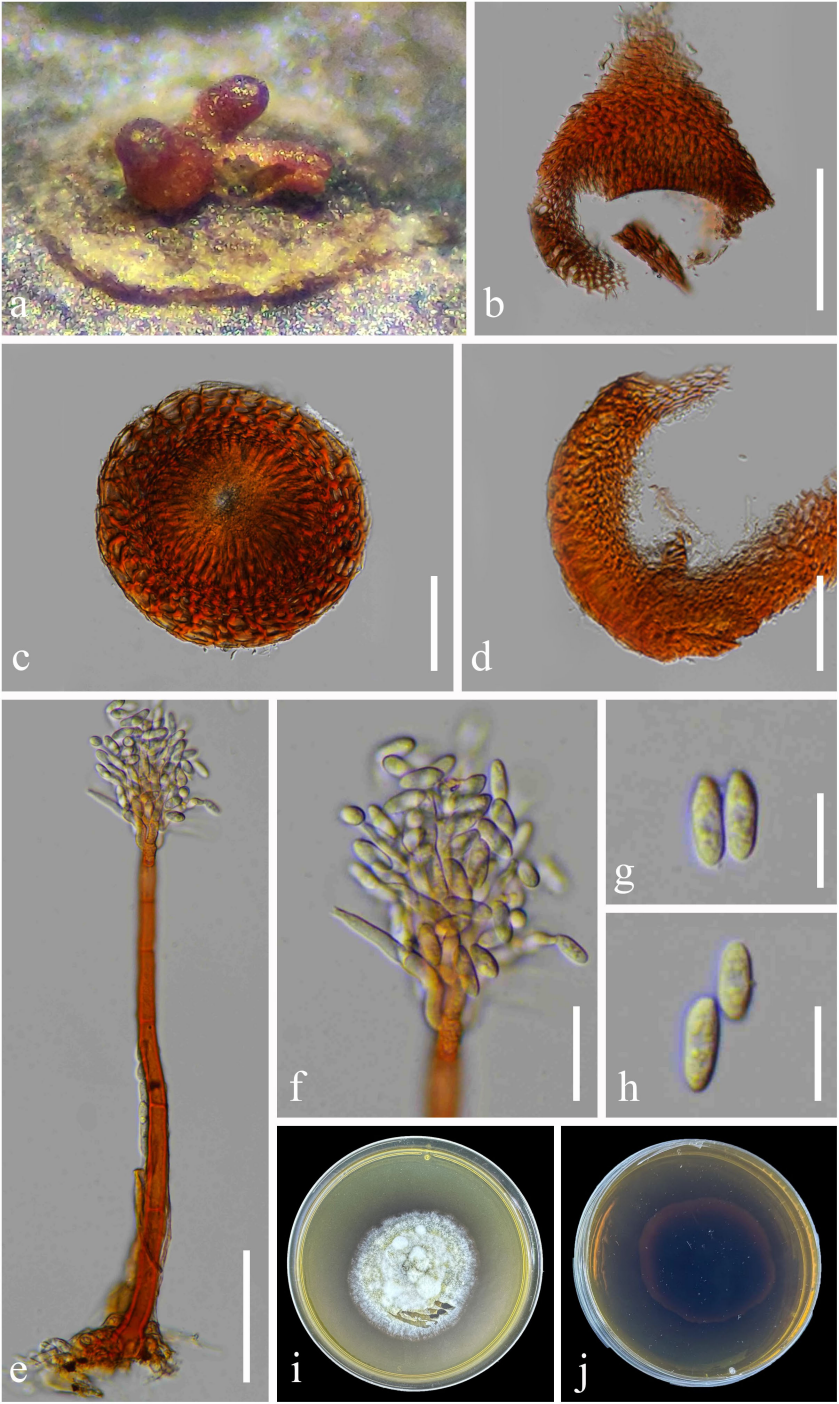
*Chaetopsina yunnanensis* (KUN-HKAS 126987, *holotype*). **(A)** Hyphomycetous colonies associated with ascomata (in group) on pseudostomata on submerged branch. **(B)** Pyriform ascoma with a conical apex. **(C)** Upper view of ascoma with pore-like opening ostiole. **(D)** Vertical section of peridium. **(E, F)** Conidiophores and conidiogenous cells bearing conidia. **(G, H)** Conidia. **(I, J)** Culture characteristics on PDA (**I** = from up-front, **J** = down-reverse). Scale bars: **(B)** = 100 μm, **(C–E)** = 50 μm, **(F)** = 20 μm, and **(G, H)** = 10 μm. PDA, potato dextrose agar.

Index Fungorum number: IF901629, **Figure 4**

Etymology: The name reflects the location of Yunnan where the holotype was collected.

Holotype: KUN-HKAS 126987

*Saprobic* on an unidentified branch, submerged in a freshwater habitat. **Sexual morph**: *Ascomata* perithecial, raised from pseudostomatic, visible as orangish to reddish brown, solitary or in groups (2–3 ascomata), superficial, scattered, associated with *Chaetopsina*-like asexual morph, ovoid to obpyriform, with an acutely conical apex, ostiolate with pore-like opening, soft and freshly, shiny, collapsing when dry. *Peridium* 20–50 µm ( $\bar{x}$ = 38 µm, n = 20) thick in vertical section, composed of thick-walled, orangish cells, of *textura angularis* to *textura prismatica* or *epidermoidea*, becoming paler and slightly elongated toward interior. *Asci and ascospores* were not observed. **Asexual morph**: *Conidiophores* 200–310 µm long, 5–11 µm wide, broader at bulbose base with 8–10 µm wide, erect, macronematous, mononematous, straight to slightly curved, 5–8-septate, thick-walled, smooth, orangish brown to reddish brown, paler toward apex, fertile at above half. *Fertile region* yellow to orangish brown comprising loosely and regularly arranged penicillate, smooth-walled, 2-septate branches bearing densely aggregated conidiogenous cells, arranged in several whorls. *Conidiogenous cells* 6–8 × 4–6 µm ( $\bar{x}$ = 7 × 4.5 µm, n = 20), discrete, ampulliform, phialidic, hyaline. *Conidia* 8–11 × 5–7 µm ( $\bar{x}$ = 9.5 × 6 µm, n = 20), cylindrical to cylindric-fusoid or ellipsoidal, hyaline, solitary, unicellular, guttulate, smooth.

*Culture characteristics*: Conidia germinating on PDA within 48 h. Colonies on PDA reaching 20 mm diam. at room temperature in natural light after 1 month. Colonies from above medium dense, circular, dull, flat to slightly raised, effuse to fairly fluffy, white to cream, edge entire, with a rough surface, slightly radiating with pale yellowish concentric ring outward colonies, sometimes with raised floccose mycelial turfs; reverse: dark reddish brown, paler at margins, with black reddish pigments produced in PDA. Sporulation not observed.

*Material examined:* China, Yunnan Province, Xishuangbanna (99°56′–101°50′E, 21°08′–22°36′N, 470–2,429.5 msl), on a branch submerged in a freshwater stream, 9 September 2022, L. Li, LILU-133 (KUN-HKAS 126987, holotype), ex-type living culture KUNCC23-12940; *ibid.*, Kunming, Yang Zonghai Lake (102°5′–103°02′E, 24°51′–24°58′N, 1,770 msl; pH of water = 8.92, temperature = 26°C, dissolved oxygen = 7.6 mg/L, purification level 3), on submerged wood, 12 July 2022, L. Li, LILU-133-1 (KUN-HKAS 126988), living culture KUNCC23-13014.

*Notes:* The nucleotide BLAST searches of ITS and LSU sequences indicated that *C. yunnanensis* sp. nov. (KUNCC23-12940) is close to *Chaetopsina* species, with LSU of 99% similarity to *C. pinicola* (CBS 136444), *Chaetopsina gautengina* (CPC 34896), and *C. pnagiana* (BRFM 3055); ITS of 97% similarity to *C. pinicola* (CBS 136444); and 96% similarity to *C. fulva* (CBS 138004) and *C. gautengina* (CPC 34896). In addition, the morphological characteristics of *C. yunnanensis* well match the concept of *Chaetopsina*, with red-pigmented upright conidiophores bearing discrete, phialidic conidiogenous cells and unicellular, cylindrical to cylindric-fusoid or ellipsoidal conidia (Kirk and Sutton, 1985; Samuels, 1985; Lechat and Fournier, 2020). The concatenated ITS and LSU phylogenetic analyses also demonstrated that the new strains KUNCC23-12940 and KUNCC23-13014 clustered within the species group of *Chaetopsina* but constituted an independent branch and sister to *C. pinicola* (CPC 21819) (**Figure 1**). The nucleotide base pair comparison between the type strain of the new species, KUNCC23-12940, and *C. pinicola* (CPC 21819) revealed 18/586 bp (3.07%) of ITS and 4/800 bp (0.5%) of LSU differences. Morphologically, *C. yunnanensis* differs from *C. pinicola* in having darker reddish-pigmented, 5–8-septate conidiophores, with penicillate fertile region at the apex and cylindric-fusoid or ellipsoidal conidia. In contrast, *C. pinicola* has medium brown, turning red-brown in 3% KOH, unbranched, verruculose, 11–15-septate conidiophores, with fertile mid region and subcylindrical, larger conidia [(11–)13–15(–17) × 2(–2.5) µm; Crous et al., 2013].

**Class** Sordariomycetes O.E. Erikss. & Winka

**Subclass** Savoryellomycetidae O.E. Erikss. & Winka

**Order** Fuscosporellales Jing Yang, Bhat & K.D. Hyde

**Family** Fuscosporellaceae Jing Yang, Bhat & K.D. Hyde

**Genus** *Parafuscosporella* Jing Yang & K.D. Hyde

***Parafuscosporella hunanensis*** L. Li, Bhat & Phookamsak, **sp. nov.**

**Figure 5:**
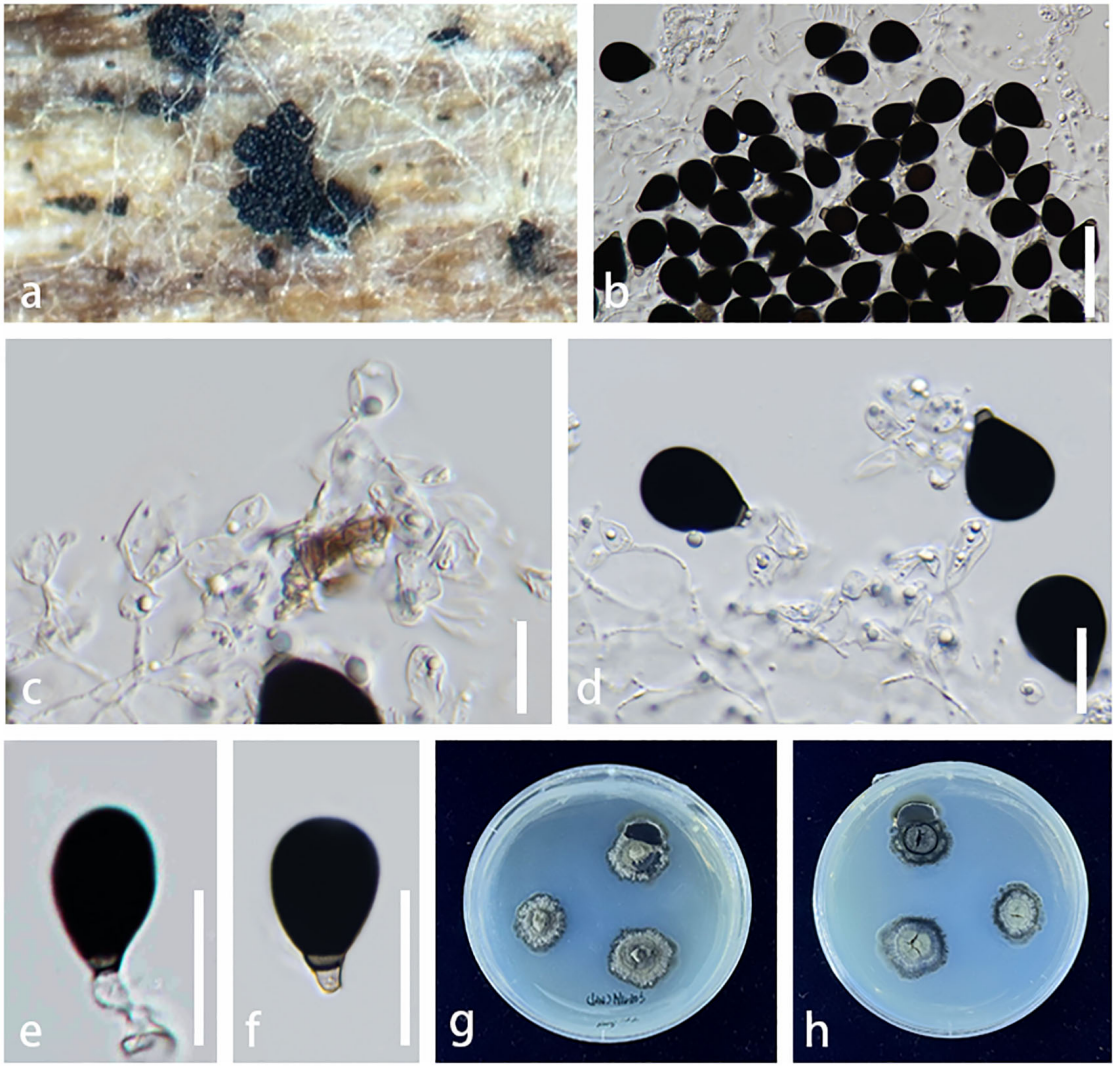
*Parafuscosporella hunanensis* (KUNCC23–13574, *holotype*). **(A)** Sporodochia on host surface. **(B)** Conidial mass with dense, inconspicuous conidiophores, embedded in gelatinous matrix. **(C)** Micronematous, branched septate conidiophores with ampulliform to doliiform conidiogenous cells. **(D)** Conidiophores and conidiogenous cells bearing conidia. **(E, F)** Conidia with a distinct basal frill derived from the distal end of the conidiogenous cell or with pale brown to hyaline protuberance. **(G, H)** Colony on PDA (**G** = from above, **H** = from below). Scale bars: **(B)** = 50 μm, **(C–F)** = 20 μm. PDA, potato dextrose agar.

Index Fungorum number: IF 902698, **Figure 5**

Etymology: The name reflects the location Hunan Province of China where the holotype was collected.

Holotype: KUN-HKAS 136260

*Saprobic* on decaying submerged wood in a freshwater stream. **Sexual morph**: Undetermined. **Asexual morph** *Colonies* on natural substrate sporodochial, scattered, sparse, visible as black, dull, soft, granular on the host surface. *Mycelium* semi-immersed to superficial, composed of septate, hyaline, and smooth hyphae. *Conidiophores* 10–35 × 3–10 µm ( $\bar{x}$ = 23 × 7 µm, n = 20), micronematous to semi-macronematous, mononematous, inconspicuous, hyaline, cylindrical, dense, erect or flexuous, branched, septate, smooth-walled. *Conidiogenous cells* 7–15 × 4–12 µm ( $\bar{x}$ = 11 × 8 µm, n = 20), holoblastic, monoblastic, integrated, terminal, determinate, hyaline to pale brown, ampulliform to doliiform, smooth-walled. *Conidia* 20–29 × 15–22 µm ( $\bar{x}$ = 26 × 19 µm, n = 20), acrogenous, obovoid to pyriform, with base truncate, dark brown to black, versicolored, paler brown at basal cell, 1-septate near the base, sometimes with pale brown to hyaline protuberance, or with a distinct basal frill derived from the distal end of the conidiogenous cell on cessation, smooth-walled.

*Culture characteristics*: Conidia germinating on PDA within 24 h. Germ tubes produced from both ends. Colonies on PDA reaching 1 mm diam. at room temperature in natural light after 3 months. Colonies from above, dense, irregular in shape, with undulate margin, dull, flat to slightly raised, velvety to felted, dark greenish gray at the margin, pale greenish gray toward the center, slightly radiated with convex ring, surface rough, with winkled folded aspect; reverse, dark greenish at the margin, pale greenish gray toward the center, radiated with black and white ring, radially furrowed at the edge; not produced pigmentation. Sporulation not observed.

*Material examined:* China, Hunan Province (108°47′–114°15′E, 24°38′–30°08′N, 500–1,500 msl), saprobic on decaying wood submerged in a freshwater stream, 26 August 2022, L. Li, LILU-203 (KUN-HKAS 136260, holotype), ex-type living culture, KUNCC23–13574; *ibid.*, LILU-203-2 (KUN-HKAS 136261), living culture KUNCC24-17774.

*Notes:* The nucleotide BLAST searches of ITS, LSU, and SSU sequences indicated that *Pa. hunanensis* sp. nov. (KUNCC23-13574) is similar to *Pa. mucosa* (MFLUCC 16-0571, type strain) with 97.73% similarity of ITS, 99.88% similarity of LSU, and 100% similarity of SSU. Multigene phylogenetic analyses demonstrated that *Pa. hunanensis* (KUNCC23-13574 and KUNCC24-17774) clustered with the type strain of *Pa. mucosa* (MFLUCC 16-0571) with high support (100% MLBS/1.00 BYPP; **Figure 2**). *Pa. hunanensis* morphologically resembles *Pa. mucosa* (MFLU 16-1980) in having obovoid to pyriform, versicolored conidia, with brown to dark brown distal cell, paler basal cell, truncate base, and septate near the basal cell. However, *Pa. hunanensis* can be distinguished from *Pa. mucosa* by the absence of jelly-like cover on sporodochia and with inconspicuous, branched, septate, cylindrical conidiophores, hyaline to pale brown, doliiform conidiogenous cells and obovoid to pyriform conidia. In contrast, *Pa. mucosa* produced sporodochia with distinct jelly-like covering, macronematous, conspicuous, cylindrical conidiophores with globose, subglobose, ellipsoidal, or clavate conidiogenous cells, and obovoid to pyriform, or ellipsoidal conidia (Yang et al., 2016). The nucleotide pairwise difference comparison between *Pa. hunanensis* (KUNCC23-13574) and *Pa. mucosa* (MFLUCC 16-0571) revealed 14/602 bp (2.32%) for ITS, 1/839 bp (0.11%) for LSU, and 0/777 bp (0%) for SSU sequences. Based on the morphological distinctions, and phylogenetic evidence, coupled with the significant nucleotide pairwise differences of ITS region, the new species *Pa. hunanensis* is introduced herein.

**Class** Sordariomycetes O.E. Erikss. & Winka

**Subclass** Savoryellomycetidae O.E. Erikss. & Winka

**Order** Pleurotheciales Réblová & Seifert

**Family** Pleurotheciaceae Réblová & Seifert

**Genus** *Pleurotheciella* Réblová

***Pleurotheciella yunnanensis*** L. Li, Bhat & Phookamsak, **sp. nov**.

**Figure 6:**
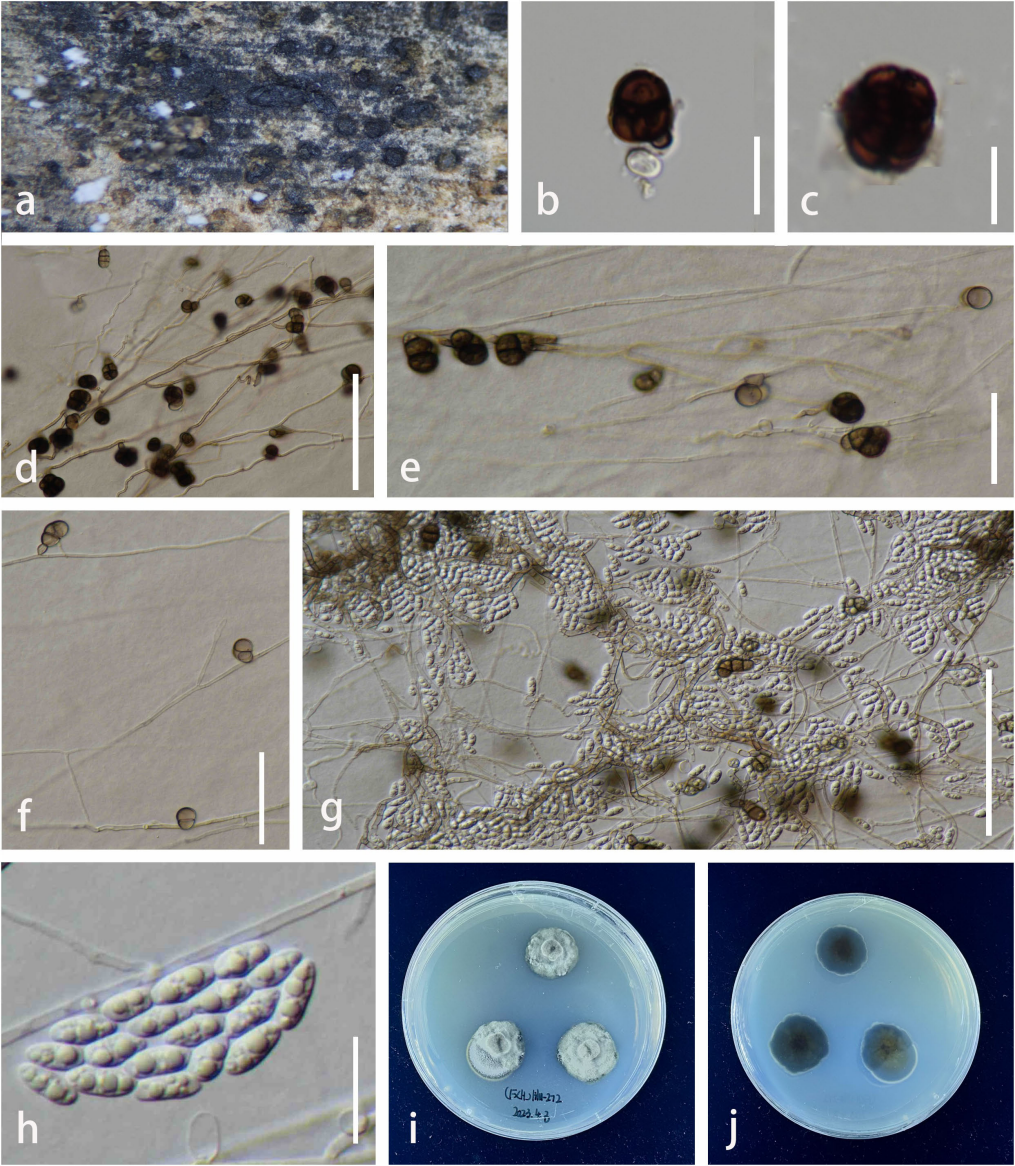
*Pleurotheciella yunnanensis* (KUN-HKAS 132016, *holotype*). **(A)** Colonies on surface of submerged wood. **(B, C)** Dark brown, muriform conidia on natural substrate. **(D–F)** Conidiogenous cells bearing muriform conidia *in vitro*. **(G)** Conidiogenous cells bearing brown, muriform conidia (Type I) and hyaline, ellipsoidal conidia (Type II) on PDA. **(H)** Hyaline conidia (Type II). **(I, J)** Colonies on PDA (**I** = from above, **J** = from below). Scale bars: **(B–E, G, H)** = 20 µm; **(F)** = 10 µm. PDA, potato dextrose agar.

Index Fungorum number: IF901610, **Figure 6**

Etymology: In reference to Yunnan Province of China, where the holotype was collected.

Holotype: KUN-HKAS 132016

*Saprobic* on decaying, submerged wood from freshwater habitats. **Sexual morph:** Undetermined. **Asexual morph:** *Colonies* on natural substrates visible as small, black, scattered, dots on the host surface. *Mycelium* immersed to partially superficial, brown to dark brown, composed of branched, septate, smooth, 2–3 µm wide, thin-walled hyphae. *Conidiophores* difficult to distinguish on the host, semi-macronematous, mononematous, sub-hyaline to brown, erect, or bent on the host surface. *Conidiogenous cells* 3–5 × 3–6 µm ( $\bar{x}$ = 4 × 4.5 µm, n = 20), holoblastic, hyaline, raised from hyphae, terminal or intermediate. *Conidia* 18–25 × 22–30 µm ( $\bar{x}$ = 21.5 × 26 µm, n = 20), varied in shape, ellipsoidal to subglobose, dark brown to black, initially forming phragmoconidia, later becoming muriform, chairoid at maturity. *In vitro:* Dimorphic, with two conidial types. Type I: *Conidiophores* 30–50 × 3–5 µm ( $\bar{x}$ = 36 × 4.5 µm, n = 20), semi-macronematous or macronematous, mononematous, hyaline to dark brown, cylindrical, septate, unbranched. *Conidiogenous cells* holo- to polyblastic, terminal, lateral, or intercalary, brown. *Conidia* 15–22 × 12–15 µm ( $\bar{x}$ = 19 × 13.5 µm, n = 20), phragmosporous to muriform, variedly shaped, brown to dark brown, subglobose to cordiform, or irregular in shape, with a protuberant hilum, initially 2–3-phragmoseptate, at maturity becoming 1–2 transverse and longitudinally dictyoseptate, sectored, leaf clover-like, brown to dark brown. Type II: *Conidiophores* reduced to conidiogenous cells. *Conidiogenous cells* 3–8 × 2–6 µm ( $\bar{x}$ = 5.5 × 5 µm, n = 20), phialidic, terminal, integrated, with minute denticles, subhyaline to pale brown, 1–2-septate, unbranched, arising in pseudo-chains. *Conidia* 7–12 × 3–5 µm ( $\bar{x}$ = 9.5 × 4 µm, n = 20), hyaline, ellipsoidal, 0–1-septate, guttulate, smooth-walled.

*Culture characteristics:* Conidia germinating on PDA within 48 h. Germ tubes produced from the basal cell. Colonies reaching 2.3 mm diam at room temperature in normal day and night light after 1 month. Colonies from above, white to pale gray, dense, slightly circular to irregular in shape, slightly raised to umbonate, rough at the surface, with wrinkled folded aspect, undulate at the edge, slightly radiating, velvety to felted; reverse, white at the margin, dark green to black in the middle, olive in the center, wrinkled folded; not producing pigmentation. Mycelium superficial to immersed in media, brown, composed of septate, branched, smooth hyphae. Sporulation on PDA after 2 weeks.

*Material examined:* China, Yunnan Province, Fuxian Lake (102°43′–102°59′E, 24°31′–24°51′N, 1,720 msl; pH of water = 8.593333, temperature = 23°C, dissolved oxygen = 7.39 mg/L, purification level 1), on decaying wood submerged in a freshwater lake, 14 August 2022, L. Li, LILU-272 (KUN-HKAS 132016, holotype), ex-type living culture KUNCC23-13328; *ibid*., Yang Zonghai Lake (102°5′–103°02′E, 24°51′–24°58′N, 1,770 msl; pH of water = 8.92, temperature = 26°C, dissolved oxygen = 7.6 mg/L, purification level 3), 12 July 2022, L. Li, LILU-136 (KUN-HKAS 132017), living culture KUNCC23-13682.

*Notes:* Based on the nucleotide BLAST search, *Pl. yunnanensis* (KUNCC23-13328) is similar to *Pl. dimorphospora* (KUMCC 20-0185) with 96.21% similarity of the ITS, 98.88% similarity of LSU, 99.44% similarity of SSU, and 92.29% similarity of *RPB2*. The multigene phylogenetic analyses confirmed the phylogenetic affinity of the new species (KUNCC23-13328 and KUNCC23-13682) as a member of *Pleurotheciella* by nested with *Pl. dimorphospora* and *Pl. saprophytica* forming a well-resolved subclade among other species within the *Pleurotheciella* (**Figure 3**). The nucleotide pairwise comparison between *Pl. yunnanensis* (KUNCC23-13328) and *Pl. saprophytica* (MFLU 17-0915) revealed 16/464 bp (3.4%) of ITS, 7/804 bp of LSU (0.9%), 0/813 bp of SSU (0%), and 40/781 bp of *RPB2* (5%) differences. *Pl. yunnanensis* shares similar morphology with *Pl. dimorphospora* in having two types of conidial morphology *in vitro*. However, they differ by phragmoconidia comprising 2–3-septate and leaf clover-like, brown to dark brown dictyoconidia. In contrast, *Pl. dimorphospora* mainly produced multi-septate, sectored, ellipsoidal to subglobose dictyoconidia (Boonmee et al., 2021). Furthermore, *Pl. dimorphospora* was found in terrestrial habitats, whereas *Pl. yunnanensis* has been found in aquatic habitats. *Pl. yunnanensis* differs from *Pl. saprophytica* by the lack of cylindrical or apically tapering sympodial, conspicuously denticulate conidiogenous cells and subcylindrical to obovoidal, obtuse conidia with a rounded apex and tapering base (Luo et al., 2018). However, both species were isolated from submerged wood in a freshwater environment in Yunnan Province, China. Interestingly, most *Pleurotheciella* have been found in aquatic environments, except for *Pl. dimorphospora*. Details on habitats, hosts, and distributions of *Pleurotheciella* are provided in **Table 4**. Furthermore, *Pl. yunnanensis* fits well with the generic concept of *Pleurotheciella* by sharing similar morphological characteristics as polyblastic, denticulate conidiogenesis, subhyaline conidiophores, and hyaline, ellipsoidal to ellipsoidal-fusiform, and aseptate to multi-septate conidia. Detailed morphological characteristics of all known *Pleurotheciella* species are also provided in **Table 5**.

**Table 4:** Distribution, habitats, and hosts of *Pleurotheciella* species.

Species name	Host	Habitats	Distribution	Reference
*Pleurotheciella aquatica*	Submerged wood	Freshwater	Dulong River, Yunnan, China	Luo et al., 2018
*Pleurotheciella centenaria*	Submerged wood	Freshwater	Ontario, Canada	Réblová et al., 2012
*Pleurotheciella dimorphospora*	Dead wood	Terrestrial	Kunming, Yunnan, China	Boonmee et al., 2021
*Pleurotheciella erumpens*	Submerged wood	Freshwater	Ariége, France Asturias, Spain	Réblová et al., 2020
*Pleurotheciella fusiformis*	Submerged wood	Freshwater	Dulong River, Yunnan, China	Luo et al., 2018
*Pleurotheciella ganzhouensis*	Submerged wood	Freshwater	Ganzhou, Jiangxi, China	He et al., 2024
*Pleurotheciella guttulata*	Submerged wood	Freshwater	Qiubei, Yunnan, China	Luo et al., 2018
*Pleurotheciella irregularis*	Submerged wood	Freshwater	Nanchang, Jiangxi, China	He et al., 2024
*Pleurotheciella krabiensis*	Submerged wood	Freshwater	Krabi, Thailand	Hyde et al., 2018
*Pleurotheciella lunata*	Submerged wood	Freshwater	Dulong River, Yunnan, China	Luo et al., 2018
*Pleurotheciella nilotica*	Submerged wood	Freshwater	Sohag, Egypt. Nakhon Phanom, Thailand Qiubei, Yunnan, China	Abdel-Aziz et al., 2020 Shi et al., 2021
*Pleurotheciella rivularia*	Submerged wood	Freshwater	Ariége, France	Réblová et al., 2012
*Pleurotheciella saprophytica*	Submerged wood	Freshwater	Dulong River, Yunnan, China	Luo et al., 2018
*Pleurotheciella submersa*	Submerged wood	Freshwater	Dulong River, Yunnan, China	Luo et al., 2018
*Pleurotheciella sympodia*	Submerged wood	Freshwater	Nakhon Phanom, Thailand Chiang Mai, Thailand Phayao, Thailand	Shi et al., 2021
*Pleurotheciella tropica*	Submerged wood	Freshwater	Phang Nga, Thailand	Hyde et al., 2018
*Pleurotheciella uniseptata*	Submerged wood	Freshwater	Ontario, Canada Dulong River, Yunnan, China	Réblová et al., 2016
*Pleurotheciella verrucosa*	Submerged wood	Freshwater	Jian, Jiangxi, China	He et al., 2024
***Pleurotheciella yunnanensis***	**Submerged wood**	**Freshwater**	**Fuxian Lake and Yangzonghai Lake, Yunnan, China**	**This study**

The new species is indicated by black bold.

**Table 5:** Morphological characteristics of *Pleurotheciella* species (asexual morphs).

Species name	Conidiophores	Conidiogenous cells	Conidia	References
*Pleurotheciella aquatica*	28–46 × 4–5 μm, cylindrical, dark brown at below half, pale brown to hyaline at above half, straight or slightly sinuous, with a terminal node of denticles	Polyblastic, integrated, terminal, cylindrical or tapering toward tip, hyaline to subhyaline	15.5–17.5 × 3–4 μm, hyaline, broadly lunate to suballantoid, obtuse and tapering at both ends, 0–3-septate, mostly 1-septate with an inconspicuous central septum	Luo et al., 2018
*Pleurotheciella dimorphospora*	On substrate: 9–15(–21) × 2–4 μm, macronematous, mononematous, subhyaline to brown, septate, unbranched to branched *In vitro*: Type I: 4.5–6 × 2.5–4 μm, semi-macronematous or macronematous, mononematous, cylindrical, hyaline to brown, straight or flexuous, septate, unbranched Type II: reduced to conidiogenous cells	Holoblastic, terminal, integrated Holoblastic, terminal or intercalary, integrated, brown 6–8 × 2–3 mm, holoblastic, terminal, hyaline, ellipsoidal, aseptate, unbranched	(16.5–)20–27(–32) × (15–)20–25(–28) mm, dark brown to black, muriform, variedly shaped, ellipsoidal, subglobose, or irregular in shape, sectored, inconspicuously septate 20–29 × 14–19 mm, brown, muriform, varied in shape, subglobose to cordiform, or irregular in shape, with a protuberant hilum 8.5–10.8 × 4–5 mm, hyaline, ellipsoidal, 0–1-septate, 2-guttulate, smooth-walled	Boonmee et al., 2021
*Pleurotheciella fusiformis*	34–40 × 2.5–3.5 μm, hyaline, erect, arising directly on substrate, usually with a terminal node of denticles, or sometimes extending through the original node with a new extension of the conidiophore	Polyblastic, integrated, terminal, cylindrical or tapering toward tip, sympodial extended, denticulate, with conspicuous denticles, hyaline to subhyaline near base, hyaline toward apex	16–18 × 3–4 μm, hyaline, broadly lunate to suballantoid, obtuse and tapering at both ends, 0–1-septate with an inconspicuous central septum, often with 2 large guttules in each cell	Luo et al., 2018
*Pleurotheciella ganzhouensis*	9.9–41.9 × 2.2–3.7 μm, macronematous, mononematous, cylindrical, erect or slightly curved, the top slightly swollen with denticulate conidiogenous loci	Polyblastic, integrated, terminal, hyaline, cylindrical, or verrucose, forming conidia sympodially on cylindrical denticles	14.4–19.4 × 2.5–3.3 μm, hyaline, capsule-shaped, fusiform, cylindrical or subclavate, guttulate, round and tapering at both ends, one end is usually sharper, 1-septate	He et al., 2024
*Pleurotheciella guttulata*	188–310 × 3–4 μm, cylindrical, hyaline, straight or slightly sinuous, arising directly on substrate, with a terminal node of denticles	Polyblastic, integrated, terminal, hyaline, cylindrical or tapering toward tip, sympodial	17–19 × 4–5 μm, hyaline to grayish, subcylindrical, fusiform to slightly obovoid, rounded at both ends, aseptate, smooth-walled	Luo et al., 2018
*Pleurotheciella irregularis*	50–110 × 2.6–3.5 μm, macronematous, mononematous, hyaline, cylindrical, mostly tapering toward the apex, mostly curved, verrucous, irregular, some with a terminal node of denticles, with verruca in the middle and upper parts	Polyblastic, integrated, terminal, hyaline, cylindrical, or verrucose, forming conidia sympodially on cylindrical denticles	24.2–33.9 × 4.2–6.4 μm, hyaline, narrowly fusiform, subclavate, guttulate, straight or slightly arcuate, pointed at one end the other round and wider in the middle, 1–3-septate, slightly constricted at the septum	He et al., 2024
*Pleurotheciella krabiensis*	240–390 × 3.3–4.8 μm, brown, septate, becoming paler toward the apex, straight or slightly curved, splaying out at the apex	Polyblastic, sympodial, pale brown or hyaline, cylindrical or tapering apex, denticulate, denticles conspicuously cylindrical	19–25 × 4.5–6 μm, hyaline, fusiform, subcylindrical to obovoid, or subclavate, 1-septate, often guttulate, straight or slightly curved, obtuse at the apex, pointed at the base	Hyde et al., 2018
*Pleurotheciella lunata*	26–44 × 3.5–4.5 μm, cylindrical, dark brown at the base, pale brown to grayish toward the apex, straight or slightly sinuous, 2–7-septate, arising directly on substrate, with a terminal node of denticles	Polyblastic, integrated, terminal, subhyaline to hyaline, cylindrical or tapering toward tip, sympodial extended, denticulate, with conspicuous denticles	13–23 × 3–4 μm, hyaline to whitish gray, Broad lunate, rounded at the apex, obtuse and tapering at the base, 1-septate with an inconspicuous central septum	Luo et al., 2018
*Pleurotheciella nilotica*	23–110 × 3–4 μm, cylindrical, dark brown at the base, pale brown to grayish toward the apex, straight or slightly curved, unbranched, 1–8-septate, with a limited, terminal node of denticles, thick and smooth-walled, arising from thick, brown to dark brown hyphae (3–4 μm wide), similar to conidiophores and running parallel to the substrate surface	2–5 × 2.5–3.5 μm, polyblastic, integrated, terminal, cylindrical or tapering toward tip, pale-brown to subhyaline near base, hyaline toward apex, smooth-walled, forming conidia sympodially on conspicuous denticles	8–13 × 2–4 μm, clavate, lunate, hyaline, 1-septate with inconspicuous central septum, rounded at the apex, obtuse and tapering toward the base, smooth-walled, often with one or two guttules in each cell	Abdel-Aziz et al., 2020
*Pleurotheciella rivularia*	9.5–20 × 2.5–3(–3.5) μm, sub-hyaline, hyphae, often reduced to the conidiogenous cells, 1–2-septate, unbranched to sparingly branched	1.5 × 1 μm, integrated, cylindrical to ampulliform, subhyaline to hyaline, elongating sympodially, with 1–2 denticles	12.5–16.5(–17.5) × 4.5–5 μm, ellipsoidal to obovoidal, 0–1–2-septate, not or slightly constricted at the septa, individually hyaline to subhyaline, subhyaline in mass, smooth, thin-walled, solitary, rounded at the apical end, tapering toward the truncate base; subglobose conidia (8–12 × 6.5–8 μm), sometimes occur	Réblová et al., 2012
*Pleurotheciella saprophytica*	44–52 × 3–4 μm, cylindrical, dark brown at the base, pale brown to grayish toward the apex, straight or sinuous, arising directly on substrate	Polyblastic, integrated, terminal or intercalary, hyaline, cylindrical or tapering toward tip, sympodial extended, denticulate, with conspicuous denticles	10– 14 × 2.5–3.5 μm, hyaline, subcylindrical to obovoid, rounded at the apex, obtuse and tapering toward base, 1-septate with an inconspicuous central septum	Luo et al., 2018
*Pleurotheciella submersa*	113– 146 × 4.5–5.5 μm, erect, cylindrical, dark brown at the base, pale brown to grayish toward the apex unbranched, mostly 7-septate, arising directly on substrate	Polyblastic, integrated, terminal, cylindrical, sympodial extended, denticulate, with conspicuous denticles	25–28 × 5.5–6.5 μm, hyaline, subcylindrical, slightly curved, rounded at the apex, obtuse and tapering toward base, aseptate, often with 4 large guttules	Luo et al., 2018
*Pleurotheciella sympodia*	135–355 × 1.5–3.5 μm, synnemata erect, mid brown to dark brown, brown rigid, velvety, smooth, conidiophores splaying out or divergent at the apical part	Polyblastic, terminal, integrated, subhyaline to pale brown, cylindrical or subulate, smooth, denticulate, with several tiny sympodial denticles	22.5–29 × 4.5–6.5 μm, hyaline, clavate, straight or slightly curved, rounded at apex, tapering toward base, 1-septate, guttulate	Shi et al., 2021
*Pleurotheciella tropica*	100–250 × 4–4.8 μm, erect, dark brown at the base, becoming paler toward the apex, cylindrical, straight or slightly curved	Polyblastic, integrated, terminal, pale brown to hyaline, cylindrical, forming conidia sympodially on cylindrical denticles	16–21 × 5.5–7 μm, hyaline, narrowly obovoid or subclavate, guttulate, straight, obtuse at the apex, pointed at the base, 1-septate	Hyde et al., 2018
*Pleurotheciella uniseptata*	126–174 × 3.5–5.5 μm, straight or sinuous, cylindrical, dark brown at the base, becoming paler upward, smooth-walled or slightly granular or roughened, arising directly on substrate, usually with a terminal node of denticles, but rarely extending through the original node with a new extension of the conidiophore	Polyblastic, integrated, terminal, pale brown to subhyaline near base, hyaline toward apex, cylindrical or tapering toward tip, smooth-walled or slightly granular, forming conidia sympodially on conspicuous denticles	12.5– 15.5 × 3.5–4.5 μm, hyaline to grayish, Fusoid or slightly clavate, straight, rounded at the apex, obtuse and tapering toward base, 1-septate with an inconspicuous central septum, often with 1–2 large guttules in each cell	Luo et al., 2018
*Pleurotheciella verrucosa*	51.3–131.8 × 1.9–3.4 μm, macronematous, mononematous, dark brown at the base, becoming paler toward the apex, cylindrical, erect or slightly curved, slightly swollen at the base	Polyblastic, integrated, terminal, pale brown to hyaline, cylindrical or verrucous, forming conidia sympodially on cylindrical denticles or wart	10.2–16.9 × 2.3–4.3 μm, hyaline, narrowly fusiform, meniscus or subclavate, guttulate, pointed at one end, the other round and wide in the middle, 1-septate	He et al., 2024
***Pleurotheciella yunnanensis***	On substrate: Difficult to distinguish on host, semi-macronematous or macronematous, mononematous, sub-hyaline to brown, erect on host surface *In vitro*: Type I: 30–50 × 3–5 µm, semi-macronematous or macronematous, mononematous, hyaline to dark brown, cylindrical, septate, unbranched Type II: reduced to conidiogenous cells.	3–5 × 3–6 mm, holoblastic, hyaline, raised from hyphae, terminal or intermediate Holo- to polyblastic, terminal, lateral, or intercalary, brown 3–8 × 2–6 mm, phialidic, terminal, integrated, with minute denticles, subhyaline to pale brown, 1–2-septate, unbranched, arising in pseudochains	18–25 × 22–30 mm, varied in shape, ellipsoidal to subglobose, dark brown to black, initially forming phragmoconidia and becoming muriform, chairoid at maturity 15–22 × 12–15 mm, phragmosporous to muriform, variedly shaped, brown to dark brown, subglobose to cordiform, or irregular in shape, with a protuberant hilum, phragmoconidia comprise 2–3-septate, dictyoconidia comprise 1–2 transverse and longitudinal septa, sectored, leaf clover-like, brown to dark brown 7–12 × 3–5 mm, hyaline, ellipsoidal, 0–1-septate, guttulate, smooth-walled	This study

The new species is indicated by black bold.

## 4 Discussion

Freshwater fungi exhibit remarkable ecological diversity, playing essential roles in aquatic ecosystems. They act as decomposers, breaking down organic matter like wood and leaf litter, which contributes to nutrient cycling and energy flow. These fungi also form symbiotic relationships with aquatic plants and algae, aiding in nutrient uptake and survival. Some freshwater fungi are pathogens, impacting aquatic plants and animals, while others help control populations within ecosystems. Adapted to both flowing (lotic) and still (lentic) water, they show a variety of morphological and physiological adaptations that allow them to thrive in diverse aquatic environments. Freshwater fungi are also important as bioindicators, providing insights into water quality and ecosystem health. Overall, they are key contributors to ecosystem function, biodiversity, and the balance of freshwater habitats (Gessner et al., 2007; Krauss et al., 2011; Jones et al., 2014; Kuehn, 2016).

Sordariomycetes is the largest class of lignicolous freshwater fungi in Ascomycota, containing approximately 823 species and 298 genera (Luo et al., 2019; Calabon et al., 2022; Shen et al., 2022). In this study, three new freshwater species belonging to the subclasses Hypocreomycetidae and Savoryellomycetidae (Sordariomycetes) are introduced from Hunan and Yunnan provinces, China. The three new species, *C. yunnanensis*, *Pa. hunanensis*, and *Pl. yunnanensis*, are found inhabiting the freshwater habitats, both lotic and lentic. *C. yunnanensis* was collected in a lake (lentic) and a freshwater stream (lotic), *Pa. hunanensis* in a freshwater stream (lotic), and *Pl. yunnanensis* from a lake (lentic). These three species were isolated from submerged decaying branch and wood, indicating their roles as decomposers degrading organic matter in nutrient cycling. It is notable that species of *Chaetopsina*, *Parafuscosporella*, and *Pleurotheciella* are commonly known from aquatic habitats. However, many species such as *Chaetopsina guyanensis*, *C. saulensis*, and *Pl. dimorphospora* have also been found in terrestrial habitats (Lechat and Fournier, 2019; Boonmee et al., 2021). Hence, we speculate the species in genera *Chaetopsina* and *Pleurotheciella* tend to inhabit a wide range of environments, including submerged freshwater or wet terrestrial environments. However, all species of *Parafuscosporella* have so far been found in aquatic environments.

Similar to Tsui et al. (2016), the three new species—*C. yunnanensis*, *Pa. hunanensis*, and *Pl. yunnanensis*—may belong to the group of aquatic–terrestrial hyphomycetes (mitosporic ascomycetes) that are initially found growing on decaying plant material and capable of sporulation underwater. The freshwater fungi in this group such as *Canalisporium*, *Dactylaria*, *Dictyochaeta*, and *Sporoschisma*, were typically distinguished based on the features of their conidia, conidiophores, and the process of conidiogenesis (Tsui et al., 2016). Since *C. yunnanensis*, *Pa. hunanensis*, and *Pl. yunnanensis* exhibit characteristics similar to those described in aquatic–terrestrial hyphomycetes, they should fit well into this group. Their ability to grow in aquatic environments, along with their possible role in decaying plant material (e.g., branches, leaves, and wood), aligns with the ecological characteristics described for this group of fungi. However, further studies would be required to confirm their precise classification within the group, especially considering conidial morphology and conidiogeneses, as noted by Tsui et al. (2016).

Some freshwater fungi exhibit morphological and ecological specificity (e.g., aquatic spore morphology, attachment structures, growth form adaptation, and spore release strategies), which may display different morphological traits depending on their growth environment, and these traits are often linked to factors such as habitat type, nutrient sources, and interactions with other organisms (Suberkropp, 2011; Naranjo-Ortiz and Gabaldón, 2019). In this study, the three new species, *C. yunnanensis*, *Pa. hunanensis*, and *Pl. yunnanensis*, do not exhibit specific morphological and ecological adaptations to lotic and lentic environments, although detailed studies on their precise adaptations in these habitats are limited. The special morphological characteristics may inconspicuously occur in these three new species, which allow them to adapt to an aquatic environment. However, these three new three species were explored from decaying plant material submerged in freshwater lakes and streams. For instance, most species in *Parafuscosporella* possess a jelly-like cover on sporodochia or hyaline appendages at the base of conidia. *Pa. hunanensis* may produce a jelly-like cover; however, this characteristic may disappear during the shifting from the collection site to the laboratory, or this species may generally occur as saprobe in terrestrial and produce some ecological functions that allow its adaptation during its submerged in aquatic environments.

Nonetheless, we can infer potential adaptations based on their general ecological characteristics and roles in aquatic ecosystems. *C. yunnanensis*, *Pa. hunanensis*, and *Pl. yunnanensis* may exhibit morphological traits suited to their respective aquatic environments. In lotic systems, where water flow is continuous, these species may develop stronger attachment structures on their spores or hyphae, which help them remain anchored to substrates such as decaying wood or aquatic plants. In contrast, species in lentic environments, where the water is still, may not require such robust attachment features but may have adaptations for better spore dispersal in stagnant water. Additionally, species in flowing water (lotic systems) may possess conidia with characteristics that allow them to float or travel further, optimizing dispersal in currents. Conversely, in still water (lentic systems), these fungi may produce denser spores or develop morphology that enhances their survival and growth on submerged wood and organic matter, as they would be more reliant on localized nutrient sources (Kuehn, 2016).

The three new species may play crucial roles in nutrient cycling, particularly in wood degradation. In both lotic and lentic environments, they contribute to the breakdown of organic matter such as decomposing wood, which is an important source of nutrients for other organisms in the ecosystem. Their activity in wood degradation releases essential nutrients like nitrogen, carbon, and phosphorus back into the ecosystem, influencing nutrient cycling and supporting a wide range of aquatic life (Gulis et al., 2006, 2008). Moreover, the three new species may interact with other aquatic organisms, including bacteria and invertebrates, through their decomposition activities. The release of small organic molecules during wood degradation can attract microbial communities, facilitating a rich nutrient web. These interactions are crucial for maintaining the health and balance of aquatic ecosystems. The ecological relevance of these fungi in nutrient cycling and wood degradation in lotic and lentic systems is profound. In lotic environments, where constant water movement aids the dispersal of spores, the fungi can contribute to nutrient cycling over large areas, potentially impacting the entire aquatic food web. In lentic environments, they may play a more localized role but still significantly contribute to the recycling of nutrients trapped in stagnant water and organic matter. While studies on these species-specific interactions with aquatic organisms are limited, it is likely that their roles in wood degradation and nutrient cycling are shared across many freshwater fungi.

The species of *Chaetopsina* are distributed in different climate zones in various countries such as China (*C. fulva* as *C. beijingensis* and *C. hongkongensis*) (Goh and Hyde, 1997; Crous et al., 2014) Egypt (*C. aquatica*) (Bakhit and Abdel-Aziz, 2021), France (*C. guyanensis*, *C. pnagiana*, and *C. saulensis*) (Lechat and Fournier, 2019, 2020), Thailand (*C. penicillata*) (Bao et al., 2023), and South Africa (*C. gautengina*) (Crous et al., 2020). This genus has been reported from both freshwater and terrestrial habitats. For instance, *C. penicillata* was introduced by Samuels (1985) from terrestrial habitats in Ecuador, Jamaica, and New Zealand. Bao et al. (2023) reported *C. penicillata* from freshwater habitats in China. Through our study on *C. yunnanensis*, we noticed the *Chaetopsina* species can be segregated by their morphological characteristics, such as ascomata dimensions and perithecial wall anatomy, size, shape, and ornamentation of ascospores and conidia of the asexual morph. Furthermore, the morphological traits of our isolates closely resemble those attributed to *Chaetopsina*. These include red-pigmented erect conidiophores producing unicellular, cylindrical to cylindro-fusoid or ellipsoidal conidia. Phylogenetic analyses of a combined ITS and LSU sequence data indicated that our isolates *C. yunnanensis* (KUNCC23-12940 and KUNCC23-13014) are grouped with the species of *Chaetopsina*, forming a distinct lineage that is basal to *C. pinicola* (CPC 21819). Therefore, considering both the morphological comparisons and the phylogenetic analyses, we proposed the establishment of a new species, *C. yunnanensis*, in this study.

Meanwhile, we noticed that *C. beijingensis* has not been formally synonymized under *C. fulva* so far. While describing a new aquatic species, *C. aquatica*, from River Nile in Egypt, Bakhit and Abdel-Aziz (2021), quoting Lechat and Fournier (2019), referred that *C. beijingensis* is a synonym of *C. fulva*, the type species of the genus. Lechat and Fournier (2019) described two new species of *Chaetopsina* from Saül (French Guiana) and noted that *C. beijingensis* is similar to *C. fulva*, both morphologically and phylogenetically but did not taxonomically synonymize these taxa. In the present study, the phylogenetic analyses coupled with the comparison of their polymorphism demonstrated that *C. beijingensis* is conspecific with *C. fulva*, concurring with previous studies (Lechat and Fournier, 2019; Bakhit and Abdel-Aziz, 2021). Hence, *C. beijingensis* is formally synonymized under *C. fulva* herein.

Only ITS and LSU sequence data are available for most species of *Chaetopsina*, of which the ITS gene is currently remarkable as the sufficient phylogenetic marker in delineating the interspecific status of *Chaetopsina* (Lechat and Fournier, 2019, 2020; Bakhit and Abdel-Aziz, 2021). However, many sufficient phylogenetic markers derived from the protein-coding genes were recommended for resolving a better phylogenetic resolution of the family Nectriaceae where *Chaetopsina* does belong as well as the class Sordariomycetes [e.g., the ATP citrate lyase (*ACL1*), α-actin (*ACT*), β-tubulin (*TUB2*), calmodulin (*CMDA*), histone H3 (*HIS3*), the RNA polymerase II largest subunit (*RPB1*), the RNA polymerase II second largest subunit (*RPB2*), and translation elongation factor 1 alpha (*TEF1*-α)] (Lombard et al., 2015; Perera et al., 2023). Of these, sequences of the protein-coding genes are available for few *Chaetopsina* species such as *C. acutispora*, *C. fulva* (type species), and *C. penicillata* (Lombard et al., 2015). The limitation on molecular data of *Chaetopsina* can cause taxonomic ambiguities. We, therefore, recommend utilizing multigene phylogeny for resolving taxonomic ambiguities and also providing a better taxonomic resolution on *Chaetopsina*, expanding to Nectriaceae as well as the class Sordariomycetes, of which the sufficient phylogenetic markers based on the protein-coding genes of *Chaetopsina* should be derived.

In our studies on *Pa. hunanensis*, we noticed that the species of *Parafuscosporella* are morphologically indistinguishable. Hence, it was found necessary to understand their taxonomic boundaries using multigene phylogenetic markers. Phylogenetic affinities of species in *Parafuscosporella* have been delineated by ITS, LSU, and SSU phylogenetic markers, of which ITS region is currently recommended as a measurable gene for resolving phylogenetic relationships among species in *Parafuscosporella* (Boonmee et al., 2021; Boonyuen et al., 2021; Li et al., 2023). Nevertheless, the *RPB2* gene has always been utilized for delineating taxa in Fuscosporellaceae (Fuscosporellales) where *Parafuscosporella* accommodated, with other related orders such as Conioscyphales, Pleurotheciales, and Savoryellales in Savoryellomycetidae (Boonyuen et al., 2016; Yang et al., 2016, 2020; Wang et al., 2023). Unfortunately, the *RPB2* gene is only available for *Pa. garethii*, *Pa. pyriformis*, and *Pa. xishuangbannaensis* in the total of the known species. This may cause the insufficient phylogenetic resolution on *Parafuscosporella* correlated with the closely related genera in the family Fuscosporellaceae, extending to the correlation with taxa among the closely related orders in Savoryellomycetidae. Thus, the *RPB2* gene is recommended in further clarifying species levels of *Parafuscosporella* corresponding with other closely related genera in Fuscosporellaceae.

The confusion between *Parafuscosporella* and *Fuscosporella* was also discussed in a previous study (Yang et al., 2016). *Fuscosporella* is morphologically similar to *Parafuscosporella* but differs in the structure of conidia, which are produced in culture. *Fuscosporella* produces multi-celled, filamentous to helicoid conidia, while *Parafuscosporella* produces globose to obpyriform, uni-septate conidia in culture. The same situation exists for *Conioscypha* in that the species of *Conioscypha* are largely indistinguishable in morphology. Li et al. (2024) proposed to use the potential of phylogenetic markers to clarify their phylogenetic relationships. The single gene trees of *Conioscypha* (ITS, LSU, SSU, and *RPB2*) and combined sequence datasets (LSU-ITS, LSU-ITS-SSU, and LSU-ITS-*RPB2*) were previously obtained to compare the reliable phylogenetic markers. The results of these prior analyses demonstrated that analysis of the *RPB2* gene could provide a better phylogenetic resolution of *Conioscypha*. Therefore, the *RPB2* gene is recommended as a genetic marker for resolving phylogenetic relationships among species in *Conioscypha* (Li et al., 2024). Simultaneously, the protein-coding genes such as *TEF1-α* and *RPB2* have been recommended for clarifying the taxonomic ambiguities of various genera in Sordariomycetes due to their high utility in resolving phylogenetic relationships at multiple taxonomic levels. *TEF1-α* and *RPB2* are relatively conserved within Sordariomycetes but also exhibit enough variability to distinguish closely related species or genera. Their nucleotide sequences evolve at rates suitable for both deep and shallow phylogenetic analyses, making them useful for resolving both inter- and intra-generic relationships (Matheny et al., 2007; Lücking et al., 2020, 2021). Therefore, it is concluded that the protein-coding genes, especially the *RPB2* gene region, are necessary for resolving the taxonomic ambiguities of some genera in Savoryellomycetidae as well as Sordariomycetes, and hence, *TEF1-α* and *RPB2* should be derived for novel taxa in further study.

It is worth noting that Goh et al. (2023) synonymized *Parafuscosporella* under *Vanakripa* due to the morphological features of *V. gigaspora*, the type of *Vanakripa*, resembling *Parafuscosporella*. Based on the principle of nomenclatural priority, all nine *Parafuscosporella* species were transferred to *Vanakripa* (Goh et al., 2023). However, *V. gigaspora* as the type species of *Vanakripa* lacks molecular data to confirm their phylogenetic placement. Hence, this leads the taxonomic confusion on the phylogenetic placement of *Vanakripa*. *Vanakripa* was introduced by Bhat and Kendrick (1993), with *V. gigaspora* as the type species. The genus was considered as genus *incertae sedis* in Pezizomycotina (Wijayawardene et al., 2021), while a key to species of the genus was provided by Arias et al. (2008). *Vanakripa* has a special clavate to vermiform, hyaline, separating cells attached to the conidia (Bhat and Kendrick, 1993). The genus currently accommodated a few species lacking genetic sequences. In Goh et al. (2023), phylogenetic analyses demonstrated that *Vanakripa* formed separated clades relating to *Conioscypha* and *Parafuscosporella*. Two new species that were identified as *V. oblonga* and *V. taiwanensis* by Goh et al. (2023) formed a clade with *Parafuscosporella* in Fuscosporellaceae, whereas *V. chiangmaiensis* and *V. minutiellipsoidea* are related to *Conioscypha* in Conioscyphaceae. Goh et al. (2023) re-circumscribed morphological features of *Vanakripa* compared to *Parafuscosporella*. Based on conidial ontogeny resemblance, Goh et al. (2023) demonstrated that *Parafuscosporella* is congeneric with *Vanakripa*. Therefore, Goh et al. (2023) transferred all *Parafuscosporella* to *Vanakripa* without providing phylogenetic evidence from type studies.

In accordance with the morphological trait, many genera in Fuscosporellaceae and Conioscyphaceae are morphologically somewhat similar; however, these genera can be distinguished based on multigene phylogeny of sufficient genes. Unfortunately, *Vanakripa* formed polyphyletic clades in Conioscyphales and Fuscosporellales (Goh et al. (2023). Considering the morphology of *Vanakripa* species, Goh et al. (2023) excluded *V. chiangmaiensis* and *V. minutiellipsoidea* (clade in Conioscyphales) and some other described species from *Vanakripa* (viz., *Vanakripa chinensis*, *Vanakripa ellipsoidea*, *Vanakripa fasciata*, *Vanakripa inflata*, *Vanakripa menglaensis*, *Vanakripa parva*, and *Vanakripa rhizophorae*) due to their conidial morphology being different from *V. gigaspora*, the type species of *Vanakripa*, in producing ellipsoidal or broadly obovoid, one-celled conidia. In contrast, *V. oblonga*, *V. taiwanensis* (clade as basal of *Parafuscosporella* in Fuscosporellales in the present study), and other synonymized *Parafuscosporella* species were treated as *Vanakripa sensu stricto* due to the conidial morphology resemblance in producing septate, apiosporous, versicolored conidia, with or without the presence of a hyaline appendage at the conidial base (Goh et al., 2023). With this point of view, it is reasonable to segregate *Vanakripa* into two morphological groups. However, molecular data of *V. gigaspora*, the type species of *Vanakripa*, have not yet been derived, and/or the epitype has not yet been designated, leading to the phylogenetic uncertainty for *Vanakripa*. Hence, the treatment of *Vanakripa* in different orders may cause taxonomic confusion in *Vanakripa*, corresponding with *Parafuscosporella*. In the present study, we, therefore, tentatively place *Parafuscosporella* as a distinct genus from *Vanakripa* to avoid taxonomic confusion until the type strain of *V. gigaspora* is derived from molecular data and/or the epitype is designated for clarifying its phylogenetic affinity. We also believe that caution of synonymization should be maintained at this stage. However, if future studies could confirm that *Parafuscosporella* and *Vanakripa* are congeneric based on morphological and phylogenetic evidence, this would greatly advance the taxonomic discussion of the Fuscosporellaceae. We recognize that any changes in classification should take into account the importance of nomenclatural stability, ensuring that the naming and classification system of the family remains consistent and carefully considered within the scientific community.

It is notable that 17 of the total 18 species of *Pleurotheciella* were collected from freshwater habitats, and only *Pl. dimorphospora* is from the terrestrial environment. Furthermore, most species have been found in China and Thailand, as well as in Canada, Egypt, France, and Spain (**Table 4**). In addition, *Pl. dimorphospora* is dimorphic (with two types of conidial morphology), which can be well distinguished from other species in *Pleurotheciella* (Boonmee et al., 2021). Initially, the original authors felt that the two types of conidial morphology *in vitro* were caused by contamination in the culture. However, through single spore isolation and molecular work on both types of conidia, they confirmed that these two types of conidia are of the same species (Boonmee et al., 2021). It is interesting that our study also meets the same situation with the new species, *Pl. yunnanensis*, showcasing two-type conidial morphology *in vitro*.

Although in recent times freshwater fungi have been continuously discovered, compared to soil ecosystems, these fungi still lacked sufficient spatial and temporal resolution, especially from those environments falling in various latitudinal zones, ecosystems (such as the water column and sediments), snowclad mountains, and extreme environments like deep-sea vents (Grossart et al., 2019). Yunnan has emerged as a hotspot for lignicolous freshwater fungal research since 2015, resulting in the discovery of a number of new species and new records in some extremely varied genera such as *Acrogenospora*, *Dictyosporium*, *Distoseptispora*, *Pleurotheciella*, *Sporidesmium*, and *Sporoschisma* (Bao et al., 2020; Wan et al., 2021; Shen et al., 2022). In contrast, the lignicolous freshwater fungi in Hunan Province still remained understudied. Hyde et al. (2016) discussed the impacts of riparian vegetation, water pollution, sampling methods, and global warming on the diversity of lignicolous freshwater fungi. However, the diversity, quantitative abundance, and ecological functions of freshwater fungi, particularly their interactions with other microorganisms, remain largely speculative, unexplored, and overlooked in the current understanding of aquatic ecology and biogeochemistry (Haraldsson et al., 2018; Grossart et al., 2019). The three new species explored in the present study from freshwater lakes and streams in Hunan and Yunnan provinces will add to the species number of lignicolous freshwater fungi in China and upgrade the global species numbers of freshwater fungi.

Additionally, genomic studies have revealed that freshwater fungi contain a vast diversity of secondary metabolite pathways (El-Elimat et al., 2021). However, the genes and gene clusters involved in these metabolic processes, as well as the secondary metabolite products, remain mostly unknown (Chiang et al., 2009; El-Elimat et al., 2021). Over the last 30 years, several freshwater fungi have been subjected to chemical investigations, resulting in the isolation of 283 secondary metabolites of wide chemical diversity and a broad range of biological activities (Wang et al., 2008; Hernández-Carlos and Gamboa-Angulo, 2011; Canto et al., 2022; El-Elimat et al., 2021). El-Elimat et al. (2021) reviewed the secondary metabolites of freshwater fungi and summarized those compounds as mainly belonging to alkaloids, terpenes, polyketides, phenylpropanoids and peptides, and some unclassified secondary metabolites. Dong et al. (2009) isolated two novel naphthalene-containing compounds, colelomycerones A and B, and three known metabolites from the culture broth of an unidentified freshwater fungus YMF 1.01029, and those metabolites showed noticeable antifungal and antibacterial activities. Prabhu et al. (2018) found Greensporone C, a secondary metabolite from freshwater fungi inducing mitochondrial-mediated apoptotic cell death in leukemic cell lines. de Souza et al. (2023) investigated communities of 154 culturable freshwater fungi from Antarctic lakes and the capabilities of all cultured fungi to produce various extracellular enzymes at low temperatures and found that the most widely produced enzymes were proteases and pectinases. These active enzymes produced by freshwater fungi have various applications in biotechnological processes in industries including textile, pharmaceutical, food, detergent, and paper, as well as in bioremediation of environmental pollutants (Raghukumar, 2008; Fathima et al., 2022; de Souza et al., 2023). Unfortunately, the study on secondary metabolites of *Chaetopsina*, *Parafuscosporella*, and *Pleurotheciella* is unexplored. Therefore, further studies on secondary metabolites of freshwater fungi in *Chaetopsina*, *Parafuscosporella*, and *Pleurotheciella* will be most rewarding.

The limitations of the current study are essential for providing a balanced perspective and guiding future research. One of the key challenges encountered in this study was the difficulty in morphological differentiation of some freshwater fungal species, particularly given the high morphological plasticity within certain genera. Additionally, our sampling efforts were constrained by limited geographic coverage and sampling time, which may have affected the comprehensiveness of the data. Furthermore, genetic sequencing efforts were limited by the availability of high-quality DNA samples from all species, which constrained the depth of phylogenetic analyses. These limitations underscore the need for more extensive and targeted genetic studies, such as the use of multigene sequencing or the application of environmental DNA (eDNA) sampling techniques, which could help capture a broader diversity of fungi in various aquatic ecosystems. Future research should prioritize these approaches to better understand the full scope of freshwater fungal biodiversity and address the unresolved taxonomic and ecological questions raised in this study.

## Data Availability

The datasets presented in this study can be found in online repositories. The names of the repository/repositories and accession number(s) can be found below: https://www.ncbi.nlm.nih.gov/genbank/ ITS: OQ860234, OQ860233, OR230704, PP744554, OR234682, PP095384; LSU: PP151255, PP151256, PP744555, PP744556, PP095383, PP095381; SSU: PP744557, PP744558, PP095382, PP095385; RPB2: PP131261, PP131262. The final alignment and phylogenetic tree were registered in TreeBASE under the submission IDs: 31916 (*C. yunnanensis*), 31910 (*Pa. hunanensis*) and 31135 (*Pl. yunnanensis*) (http://www.treebase.org/ accessed on 25 December 2024).
